# *Eremanthus erythropappus* as a Source of Antiparasitic Agents: *In Vitro* and *In Vivo* Efficacy against *Schistosoma mansoni*

**DOI:** 10.1021/acsomega.5c03005

**Published:** 2025-07-09

**Authors:** Leonardo Luiz O. de Mello, Carla M. Leal, Monique C. Amaro, Allan I. Andrade-de-Siqueira, Josué de Moraes, Ademar A. da Silva Filho, Giovanni W. Amarante

**Affiliations:** † Núcleo de Identificação e Pesquisa em Princípios Ativos Naturais (NIPPAN), Faculdade de Farmácia, Departamento de Ciências Farmacêuticas, 28113 Universidade Federal de Juiz de Fora, R. José Lourenço Kelmer s/n, Campus Universitário, Juiz de Fora, MG 36036-900, Brazil; ‡ Grupo de Pesquisas em Metodologias Sintéticas (GPMS), Departamento de Química, Instituto de Ciências Exatas, Universidade Federal de Juiz de Fora, R. José Lourenço Kelmer s/n, Campus Universitário, Juiz de Fora, MG 36036-900, Brazil; § Núcleo de Pesquisa em Doenças Negligenciadas (NPDN), 92928 Universidade Guarulhos, Guarulhos, SP 07023-070, Brazil; ∥ Núcleo de Pesquisa em Doenças Negligenciadas, Instituto Científico e Tecnológico, Universidade Brasil, São Paulo, SP 08230-030, Brazil

## Abstract

Schistosomiasis, a neglected tropical disease (NTD) caused by flatworms of the genus *Schistosoma*, affects more than 250 million people globally. Praziquantel (PZQ) remains the sole therapeutic option, underscoring the need for novel treatments. This study investigates the antischistosomal activity of *Eremanthus erythropappus* DC. MacLeish (Asteraceae) extracts, and their isolated compounds are against *Schistosoma mansoni*. *In vitro* assays revealed that the rinsed extract (LEE) exhibited a significantly lower EC_50_ (18.1 μg/mL) compared to that of the ethanolic extract (EEE) (191.5 μg/mL), with no cytotoxicity observed at concentrations up to 500 μg/mL. UHPLC-ESI-MS/MS analysis annotated 16 metabolites, including sesquiterpene lactones (STLs) and pentacyclic triterpenoids. From LEE, friedelin (**7**), betulinic acid (**8**), and acacetin (**14**) were isolated, with the latter two being reported in *E. erythropappus* for the first time. Among them, only betulinic acid (**8**) demonstrated significant *in vitro* antischistosomal activity (EC_50_ = 36.8 μM, SI > 15.5), while friedelin (**7**) and acacetin (**14**) were inactive (EC_50_ > 50 μM). In *S. mansoni*-infected mice, the oral administration of betulinic acid (**8**) (400 mg/kg) reduced the worm burden by 41.9%, whereas LEE (400 mg/kg) achieved a substantial reduction in both the worm (∼86%) and egg burden (∼81%), also decreasing immature egg deposition by ∼87%. These findings highlight the promising antischistosomal potential of *E. erythropappus*, particularly LEE, warranting further studies to optimize the isolation and characterization of bioactive compounds.

## Graphical abstract

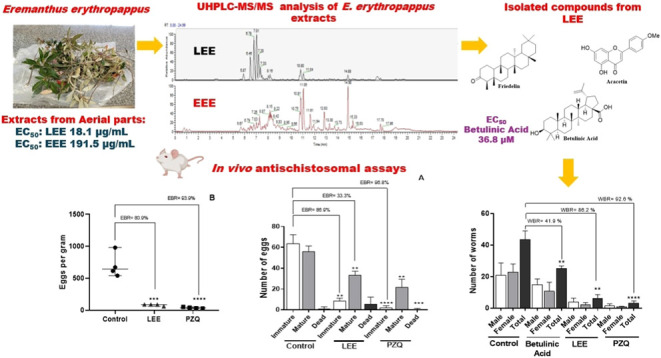

## 1 Introduction

Schistosomiasis, a debilitating parasitic disease caused by blood flukes of the genus *Schistosoma*, affects over 250 million people worldwide, with a nearly 1 billion at risk in impoverished regions of South America, Africa, and East Asia due to inadequate sanitation and contaminated water source.^1^ The disease manifests through immune-mediated granuloma formation and tissue fibrosis in target organs, positioning it among the most clinically significant helminth infections in terms of morbidity and mortality.^2,3^

Current treatment relies exclusively on praziquantel (PZQ), a pyrazine-isoquinoline derivative, favored for its low cost, oral administration, and broad efficacy against all *Schistosoma* species.^4^ However, emerging resistance, ineffectiveness against juvenile worms, and inability to prevent reinfection underscore the urgent need for new therapeutics,^4,5^ as highlighted by the WHO Neglected Tropical Diseases (NTD) Roadmap 2030, which aligns with the Sustainable Development Goals (SDGs).^6^ In this context, natural products derived from plants represent a promising alternative to the limitations of PZQ, as their diverse chemical scaffolds frequently contribute to a broad spectrum of biological activities, potentially offering novel therapeutic options.^5,7^

Among the promising sources for new antischistosomal agents is *Eremanthus erythropappus* DC. MacLeish (Asteraceae), also called *Vanillosmopsis erythropappa* Schultz-Bip, and commonly known as “candeia” and “candeia-da-serra”, being widely used in Brazilian folk medicine for its antibacterial, anti-inflammatory, antiphlogistic, and wound healing properties.^8^ The ethanolic extract (EEE) of *E. erythropappus* has pronounced anti-inflammatory and antiulcerogenic properties.^9^ Additionally, *E. erythropappus* essential oil showed antileishmanial activity against *L. amazonensis* and low cytotoxicity to mammalian host cells, supporting its potential use against cutaneous leishmaniasis.^10^ Also, preliminary studies with the dichloromethane and hexane extracts of *E. erythropappus* leaves have demonstrated *in vitro* antischistosomal activity against *S. mansoni* adult worms, highlighting its significant antiparasitic potential.^9^ Despite these promising findings, the antischistosomal potential of *E. erythropappus* remains underexplored, particularly *in vivo*.

To address this gap, we systematically evaluated the *in vitro* and *in vivo* antischistosomal activities of *E. erythropappus* extracts against *S. mansoni*, annotated metabolites in both extracts via UHPLC-MS/MS, and isolated key compounds from the most potent extract for validation in murine models. Our findings showed *E. erythropappus* as a source of antischistosomal agents, addressing both the drug development pipeline and the NTD Roadmap’s priorities.

## 2 Experimental Section

### 2.1 Plant Material

Aerial parts of *E. erythropappus* (1.2 kg) were collected at the Faculty of Pharmacy, UFJF, Juiz de Fora, MG, Brazil, in August 2022 (22° 13′ 22″ S, 44° 37′ 59″ W; SisGen number #AE32DB3). A voucher specimen of this plant was authenticated and deposited at the Leopoldo Krieger herbarium at UFJF under the number CESJ 25363.

### 2.2 Preparation of Plant Extracts

The ethanolic extract of *E. erythropappus* (EEE) was prepared by macerating the dried and pulverized aerial parts in ethanol at room temperature. After filtration, the solvent was evaporated, resulting in 12 g of crude extract, corresponding to a 10.0% yield based on dry weight.

Additionally, the rinsed extract of *E. erythropappus* (LEE) was prepared according to a previously established protocol.^11^ Aerial parts of the plant were immersed in a CH_2_Cl_2_/MeOH (1:1 v/v) solution for 25 s, at room temperature, resulting in the rupture of glandular trichomes and the extraction of their contents. The extract was then filtered and concentrated under reduced pressure, yielding 37.5 g of crude material, corresponding to a 3.1% dry weight yield.

### 2.3 UHPLC-ESI-MS/MS Analysis of LEE and EEE Extracts

Plant extracts (LEE and EEE) were analyzed using a UHPLC Dionex Ultimate 3000 system (Thermo Electron, Waltham, Massachusetts), equipped with an Agilent InfinityLab Poroshell 120 C_18_ column (100 mm × 2.1 mm, 1.7 μm, Santa Clara), applying a flow rate at 0.4 mL/min, and a mobile phase of water with 0.1% formic acid (A) and acetonitrile (B) was used. LEE and EEE extracts were analyzed using a gradient program as follows: 0–2 min, 5% B; 2–14 min, 5–98% B; 14–16 min, 98% B; and 16–20 min, 98–5% B.

In order to perform chemical characterization of the extracts, an LCQfleet mass spectrometer with electrospray ionization (ESI) source and ion trap mass analyzer was used (Thermo Electron, Waltham, Massachusetts) coupled to the UHPLC apparatus, in which the column compartment and source temperature were set at 40 and 150 °C, respectively, using high-purity nitrogen (N_2_) as the carrier gas with a flow rate of 10 L·min^–1^ and a 4 bar nebulizer gas pressure. High-purity helium (He) was used as the collision gas. MS data were acquired in both positive and negative ionization modes, using a mass range of *m*/*z* 50–1500 and a collision-induced dissociation (CID) energy of 30 eV.

The MS/MS spectral data were analyzed using the Global Natural Products Social Molecular Networking (GNPS) platform (https://gnps.ucsd.edu). For the annotation of compounds, the obtained hits in both positive and negative ionization modes were analyzed by interpretation of their fragmentation pattern. MS/MS data were converted to mzXML format using MS Convert software (https://proteowizard.sourceforge.io/), in which MZmine (version 3.28) and Xcalibur software (version 2.2) were used for data visualization and analysis of the obtained fragmentation profile and then uploaded at the GNPS platform for library comparison. GNPS molecular library search gather data can be found at https://gnps.ucsd.edu/ProteoSAFe/status.jsp?task=768bc5d42cd5483f976f2e62eacc9efb for positive mode and at https://gnps.ucsd.edu/ProteoSAFe/status.jsp?task=b1e98027b1b34cf7a5c70fc5f64ec5b0 for negative mode. GNPS parameters were set as follows: a precursor ion mass tolerance of 2.0 Da, number of minimum matched peaks of 6, a fragment ion mass tolerance of 0.5 Da, and a score threshold of 0.7.

### 2.4 Isolation and Identification of Compounds from the Rinsed Extract of *E. erythropappus* (LEE)

The LEE (20 g) extract was subjected to fractionation using vacuum liquid chromatography (VLC) with silica gel (40–63 μm) as the stationary phase. A gradient of hexane-ethyl acetate (Hex: EtOAc) mixtures, ranging from 5 to 100% ethyl acetate, was employed as the mobile phase, resulting in seven fractions. Recrystallization of fraction 1 in ethyl acetate yielded white crystals of compound **7** (70 mg). Fraction 4, after recrystallization in MeOH: EtOH (1:1, v/v), produced compound **8** (160 mg) as white crystals. Moreover, fraction 5 yielded yellow crystals of compound **14** (20 mg). The isolated pure compounds **7**, **8**, and **14** were identified as friedelin, betulinic acid, and acacetin, respectively, using ^1^H, ^13^C, and DEPT-135 nuclear magnetic resonance (NMR) spectroscopy.

NMR data for the isolated compounds were acquired at 500 MHz for ^1^H and 125 MHz for ^13^C and DEPT-135 experiments, using deuterated dimethyl sulfoxide (DMSO-*d*_6_) for betulinic acid and acacetin, and CDCl_3_ for friedelin, all at 25 °C. The spectra were recorded on a Bruker 500 Advance spectrometer (Massachusetts). Signals corresponding to deuterated solvents were used as internal standards: δH 7.26 (s), δC 77.0 ppm (t) for CDCl_3_, and δH 2.50 (quintet), δC 39.5 (sept) for DMSO-*d*_6_.

### 2.5 Animals and Parasites

The Belo Horizonte strain of *Schistosoma mansoni* was maintained in its life cycle using *Biomphalaria glabrata* snails (intermediate hosts) and female Swiss mice (final hosts).^12^ Three week old female Swiss mice (∼20 g) were obtained from Animais para Laboratório (São Paulo, Brazil). Infected snails and mice were housed under controlled conditions (25 °C, 50% humidity, and 12 h light/dark cycles) with ad libitum access to food and water.

### 2.6 *In Vitro* Antischistosomal Assays

Adult *S. mansoni* worms were harvested via portal perfusion 49 days postinfection and cultured in RPMI 1640 medium supplemented with 10% fetal bovine serum, 100 IU/mL of penicillin, and 100 μg/mL of streptomycin (37 °C, 5% CO_2_).^11,13^ Paired male and female worms were exposed to *E. erythropappus* extracts (LEE, EEE) or isolated compounds at varying concentrations. Each treatment was tested in triplicate in three independent experiments. Worm viability was monitored microscopically over 24–72 h. Worm viability was assessed based on motility; worms that remained immobile for at least 2 min were considered dead.^14^ No tegumental alterations were observed by light microscopy. *In vitro* oviposition was not monitored due to frequent separation of worm pairs during incubation.

### 2.7 *In Vivo* Antischistosomal Assays

Mice were percutaneously infected with 80 *S. mansoni* cercariae and treated 49 days postinfection (adult worm stage). Animals were randomized into four groups (*n* = 5/group) to receive the following treatments orally: LEE (400 mg/kg), betulinic acid (**8**) (400 mg/kg), praziquantel (PZQ, 400 mg/kg, positive control), or vehicle (2% ethanol in saline, negative control).^15,16^ Dosages followed established protocols for schistosomiasis drug discovery.^17^ At 63 days postinfection, mice were euthanized (CO_2_), and worms were recovered by mesenteric perfusion. Worm burden reduction was calculated after sexing,^18,19^ and fecal egg counts were performed as previously described.^11,20,21^ The study complied with ARRIVE guidelines, including randomization to minimize bias.

### 2.8 Cytotoxicity in Mammalian Cells

Vero cell viability was assessed via MTT assay.^22,23^ Cells (2 × 10^3^/well) were incubated with extracts (LEE, EEE) or compounds (friedelin, betulinic acid, acacetin) for 72 h (37 °C, 5% CO_2_). The selectivity index (SI) was calculated by the ratio of the 50% cytotoxic concentration obtained on cells (CC_50_) and 50% effective concentration determined on adult schistosomes (EC_50_).^14,24^

### 2.9 Statistical Analysis

All data were analyzed using GraphPad Prism 8.0. Dose–response curves (95% CI) determined the EC_50_ values. Group comparisons used the Kruskal–Wallis test (*P* < 0.05).

## 3 Results and Discussion

### 3.1 LEE and EEE Exhibit *In Vitro* Antischistosomal Activities

The *in vitro* evaluation of *E. erythropappus* extracts revealed pronounced differences in antischistosomal activity between the rinsed extract (LEE) and the ethanolic extract (EEE) of *E. erythropappus*. The rinsed extract (LEE) was prepared in three independent batches using the same standardized extraction protocol, and its efficacy was reproducible across all experiments. The EC_50_ values obtained were accompanied by 95% confidence intervals, demonstrating consistency between batches.

LEE demonstrated concentration- and time-dependent mortality against adult *S. mansoni* worms, achieving 100% lethality within 24 h at 200 μg/mL and 72 h at 25 μg/mL (Figure 1A,B). In contrast, EEE induced mortality only at the highest tested concentration (200 μg/mL; Figure 2A,B). Quantitative assessment further highlighted LEE’s superior efficacy, with an EC_50_ of 18.1 μg/mL compared to 191.5 μg/mL for EEE. Importantly, LEE exhibited no cytotoxicity (CC_50_ > 500 μM) against mammalian cells and displayed a high selectivity index (SI > 27.6) (Table 1), underscoring its potential as a safe and effective antischistosomal agent.

**Figure 1:**
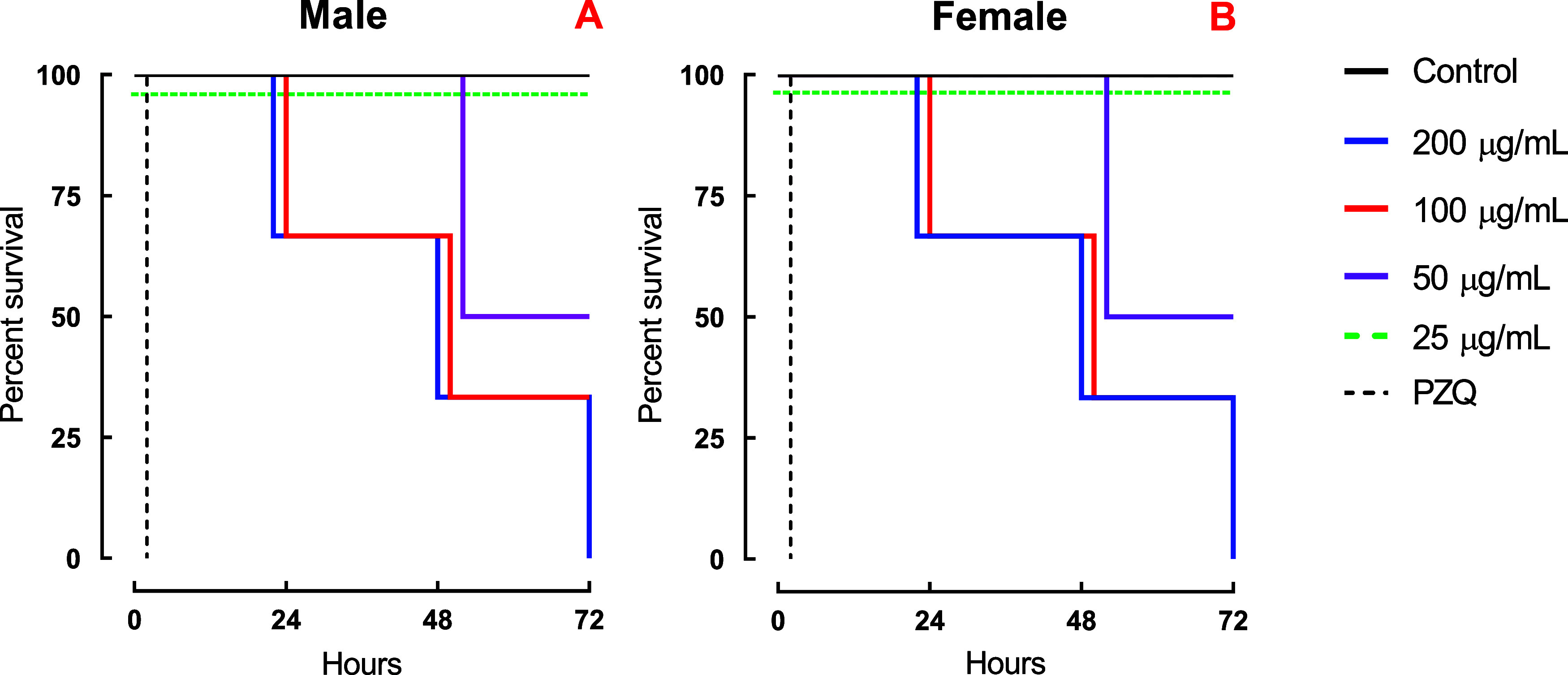
*In vitro* schistosomicidal effects of LEE extract on *S. mansoni* adult worms. Male (A) and female (B) parasites were observed for a duration of 72 h, and the outcomes are presented as the percentage of survival based on Kaplan–Meier survival curves. The average viability values were calculated from a minimum of three separate experiments (*n* = 3). Control: 0.5% DMSO in RPMI 1640 medium. PZQ: 2 μM (0.62 μg/mL).

**Figure 2:**
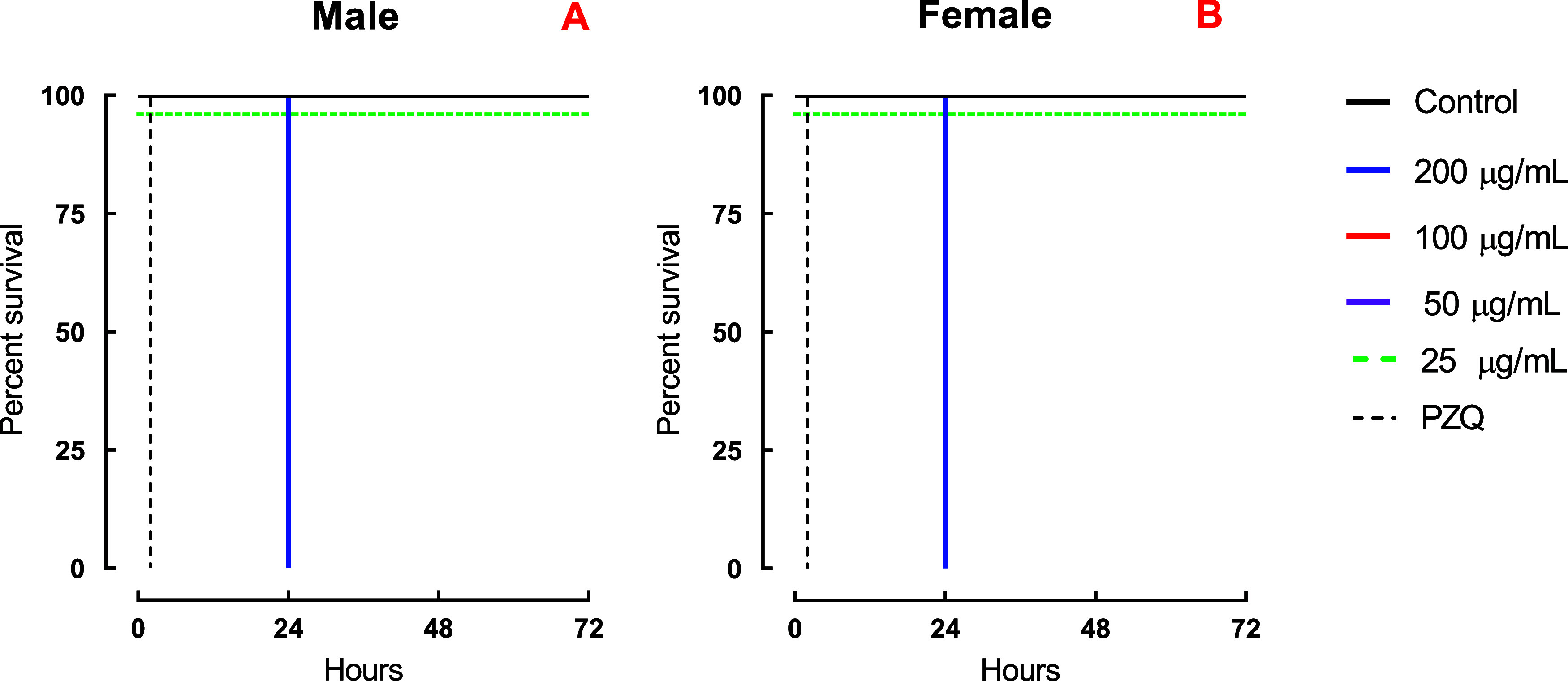
*In vitro* schistosomicidal effects of the EEE extract on *S. mansoni* adult worms. Male (A) and female (B) parasites were observed for a duration of 72 h, and the outcomes are presented as the percentage of survival based on Kaplan–Meier survival curves. The average viability values were calculated from a minimum of three separate experiments (*n* = 3). Control: 0.5% DMSO in RPMI 1640 medium. PZQ: 2 μM (0.62 μg/mL).

**Table 1:** Effective (EC_50_) and Cytotoxic (CC_50_) Concentrations of *E. erythropappus* Extracts and Compounds^a^

	*S. mansoni*	Vero cells	
samples	EC_50_	CC_50_	SI
LEE	18.1 [15.3–20.9]^b^	>500^b^	>27.6
EEE	191.5 [184.3–198.7]^b^	418.4 [386.7–450.1]^b^	2.1
friedelin (**7**)	>50^c^	ND	ND
betulinic acid (**8**)	36.8 [33.9–39.7]^c^	>500^c^	>15.5
acacetin (**14**)	>50^c^	ND	ND
praziquantel	0.8 [0.7–0.9]^c^	>500^c^	>625

a EC_50_: Effective concentration 50% based on mortality of *S. mansoni* adult worms. CC_50_: Cytotoxic concentration 50% against Vero cells after 72 h incubation. SI: selectivity Index.

b μg/mL.

c μM. Cytotoxicity activity was assessed using the MTT assay. The SI values were calculated by dividing CC_50_ values obtained on Vero cells with EC_50_ values determined on schistosomes. Values are calculated from three experiments, and each experiment was performed with three replicates. The 95% confidence interval is in square brackets.

These results align with prior studies reporting motility loss in schistosomes treated with *E. erythropappus* extracts,^8^ though the enhanced activity of LEE suggests its extraction protocol may better preserve bioactive constituents. Notably, LEE’s potency compares favorably with other bioactive plant extracts,^25–27^ such as *Pothomorphe umbellata* (EC_50_ = 9.2 μg/mL) and *Acmella oleracea* (EC_50_ = 32.6 μg/mL),^21,28^ positioning it as a promising candidate for further development.

### 3.2 UHPLC-ESI-MS/MS Analysis of LEE and EEE Extracts

UHPLC-ESI-MS/MS analysis was employed in both positive (Figure S1) and negative ionization (Figure S2) modes to annotate compounds in *E. erythropappus* rinsed (LEE) and ethanolic (EEE) extracts. Therefore, 16 metabolites (**1–16**) were annotated through spectral data analyses using the Global Natural Products Social Molecular Networking (GNPS) platform (Figures 3, S3–S18 and Table 2). Spectral hits were further manually verified for their fragmentation patterns through MS/MS data interpretation and literature comparison, where a discussion of the distinguishable set of metabolites between the extracts was mainly summarized.

**Figure 3:**
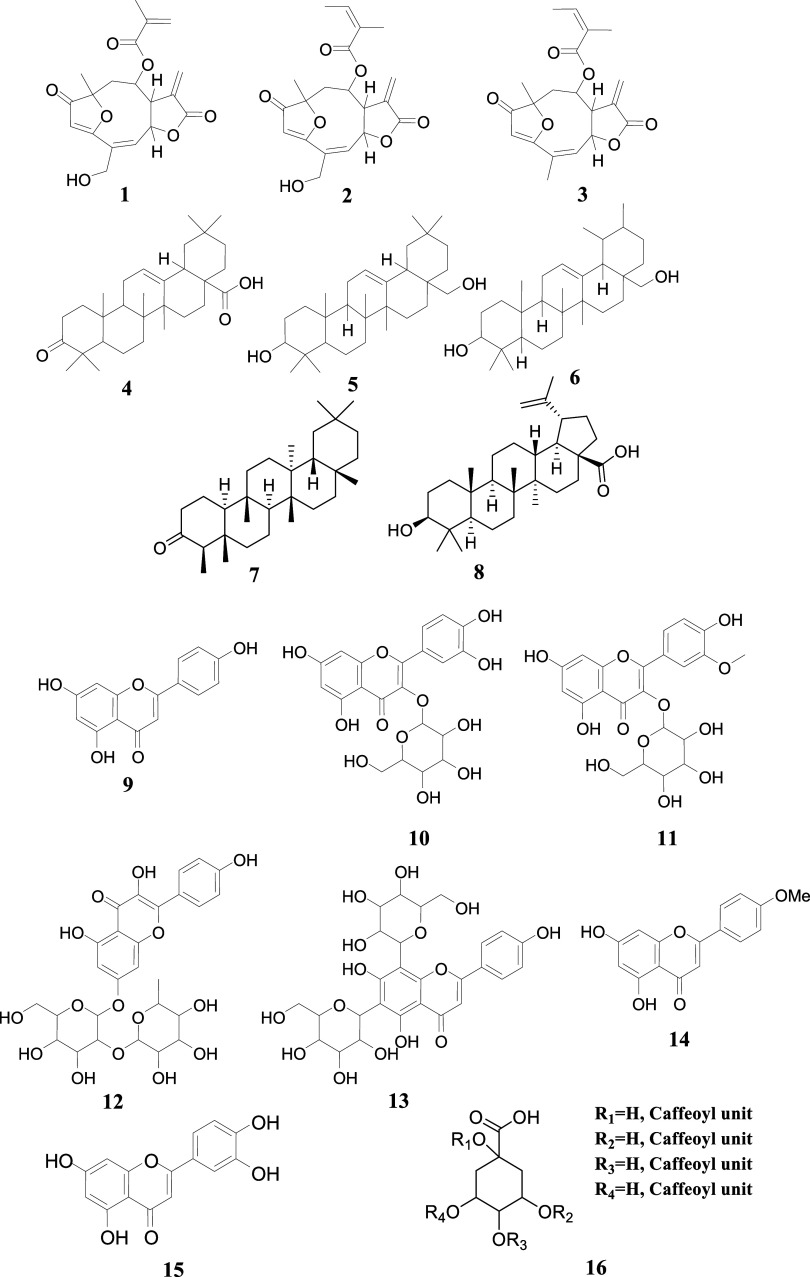
Annotated compounds goyazensolide (**1**), centratherin (**2**), lychnopholide (**3**), oleanonic acid (**4**), erythrodiol (**5**), uvaol (**6**), friedelin (**7**), betulinic acid (**8**), apigenin (**9**), isoquercetin (**10**), isorhamnetin-3-*O*-galactoside (**11**), kaempferol-7-*O*-neohesperidoside (**12**), vicenin-II (**13**), acacetin (**14**), luteolin (**15**), and di-*O*-caffeoylquinic acid (**16**) from *E. erythropappus* extracts by UHPLC-ESI-MS/MS. The proposed compounds were annotated according to the interpretation of MS^1^ and MS^2^ spectra and literature. For the determination of the stereochemistry, it was not possible to distinguish between isomers. For this reason, stereochemistry was not represented, except for friedelin (**7**), betulinic acid (**8**), and acacetin (**14**), whose structures were confirmed by NMR. Compound **16** is related to possible di-*O*-caffeoylquinic acid derivatives, where the substitution pattern might be R_1_ = caffeoyl unit, R_2_ = caffeoyl unit, R_3_=H, R_4_=H (**1,3-di-** ***O*** **-caffeoylquinic acid**); R_1_ = H, R_2_ = H, R_3_ = caffeoyl unit, R_4_ = caffeoyl unit (**4,5-di-** ***O*** **-caffeoylquinic acid**); R_1_ = H, R_2_ = caffeoyl unit, R_3_=H, R_4_ = caffeoyl unit (**3,5-di-** ***O*** **-caffeoylquinic acid**); R_1_ = H, R_2_ = caffeoyl unit, R_3_ = caffeoyl unit, R_4_=H (**3,4-di-** ***O*** **-caffeoylquinic acid**).

**Table 2:** Annotated Compounds from *E. erythropappus* Extracts (LEE and EEE) by the UHPLC-ESI-MS/MS Analysis

*E. erythropappus* extract	[M + H]^+^ (*m*/*z*)	[M – H]^−^ (*m*/*z*)	molecular formula	MS/MS (MS^2^)	proposed compound^a^	references
LEE	361		C_19_H_20_O_7_	343, 275, 257, 229, 211, 201, 179, 133	goyazensolide (**1**)	^30^
LEE	375		C_20_H_22_O_7_	356, 275, 256, 229, 211, 199	centratherin (**2**)	^30^
LEE/EEE	359		C_20_H_22_O_6_	330, 313, 276, 259, 241, 230, 212, 185	lychnopholide (**3**)	^30^
LEE	455		C_30_H_46_O_3_	437, 419, 409, 395, 367, 323	oleanonic acid (**4**)	^33^
LEE	443		C_30_H_50_O_2_	425, 411, 396, 385, 313, 237, 197	erythrodiol (**5**)	^33^
LEE/EEE	443		C_30_H_50_O_2_	425, 396, 385, 367, 351, 313, 208	uvaol (**6**)	^33^
LEE/EEE	427		C_30_H_50_O	409, 381, 368, 339	friedelin (**7**)	^36^
LEE/EEE	457		C_30_H_48_O_3_	439, 421, 393, 369, 325, 313	betulinic acid (**8**)	^33^
LEE/EEE		269	C_15_H_10_O_5_	240, 227, 210, 197, 181, 168, 149, 151, 83	apigenin (**9**)	^62^
EEE		463	C_21_H_20_O_12_	301, 271, 257, 161, 151	isoquercetin (**10**)	^63^
EEE		477	C_22_H_22_O_12_	315, 300, 285, 271, 256, 242, 216, 190, 151	isorhamnetin-3-*O*- galactoside (**11**)	^64^
LEE/EEE		593	C_27_H_30_O_15_	447, 285, 256, 240, 212	kaempferol-7-*O*- neohesperidoside (**12**)	^40^
EEE		593	C_27_H_30_O_15_	502, 473, 413, 383, 353, 311, 297	vicenin-II (**13**)	^65^
LEE/EEE		283	C_16_H_12_O_5_	268	acacetin (**14**)	^42^
LEE/EEE		285	C_15_H_10_O_6_	257, 243, 241, 217, 199, 185, 174, 168	luteolin (**15**)	^66^
LEE/EEE		515	C_25_H_24_ O_12_	353, 335, 291, 191, 178	di-*O*-caffeoylquinic acid (**16**)	^43^

a Level 2 annotation confidence corresponding to a probable structure based on the library spectrum matching and diagnostic evidence.^44^

Sesquiterpene lactones (STLs) are frequently reported in *E. erythropappus* and other *Eremanthus* species, possessing remarkable antiparasitic activity.^29^ Among STLs, certain furanoheliangolide-type STLs appeared as distinguishing metabolites between LEE and EEE extracts. The compounds at *m*/*z* 361 [M + H]^+^ and *m*/*z* 375 [M + H]^+^ were annotated as goyazensolide (**1**) and centratherin (**2**), respectively (Table 2, Figures S3 and S4). Fragment ions were of a characteristic fragmentation pattern reported for those sesquiterpenoids, such as an initial neutral loss of H_2_O (−18 Da) [M + H–H_2_O]^+^, suggestive of a hydroxyl group at C-15 attributed to fragment ions at *m*/*z* 343 in goyazensolide (**1**) and *m*/*z* 357 in centratherin (**2**), followed by the C-8 ester group elimination [M + H–H_2_O-R-CO_2_H]^+^ of the precursor ion, giving the same fragment at *m*/*z* 275, which suggests different ester groups from (**1**) (C_4_H_6_O_2_) (−86 Da) and (**2**) (C_5_H_8_O_2_) (−100 Da). The following fragments at *m*/*z* 257 and *m*/*z* 229 were attributed to consecutive losses of H_2_O (−18 Da) and CO (−28 Da) units, respectively.^30^ Lychnopholide (**3**), another furanoheliangolide lactone, was also annotated in *E. erythropappus* (Table 2). Precursor ion at *m*/*z* 359 [M + H]^+^ followed a characteristic pattern of fragmentation of such metabolites, involving the direct [M + H-R-CO_2_H]^+^ elimination of the protonated molecule leading to a fragment at *m*/*z* 259 (C_5_H_8_O_2_) (−100 Da), suggestive of a C-8 ester group (Figure S5). Subsequent fragment ions correspond to consecutive losses of H_2_O (−18 Da) at *m*/*z* 241 and CO (−28 Da) units at *m*/*z* 213 and *m*/*z* 185, likewise observed for previously described goyazensolide and centratherin.^30^ Similar to other Brazilian Vernonieae tribe species, these STLs were previously described in *E. erythropappus*.^31^ However, apart from compound (**3**), which was annotated in both LEE and EEE extracts, the obtained data indicated that compounds (**1**) and (**2**) were exclusively present in the LEE extract.

Among the oleanoids, oleanonic acid (**4**) showed similar attributions to the reported literature,^32^ therefore making it possible to annotate oleanonic acid *m*/*z* 455 [M + H]^+^ in the LEE extract. A fragment ion due to water loss from the precursor ion [M + H–H_2_O]^+^ at *m*/*z* 437 (Figure S6) was readily observed in what is believed to be C-3 substituent removal leading to alkene formation, as seen for other oleanane-type pentacyclic triterpenes.^33,34^ The resulting alkene may undergo a second water loss from the [M + H–H_2_O]^+^, giving fragment ion at *m*/*z* 419 [M + H–H_2_O–H_2_O]^+^, leading to an acylium ion formation at C-28, which further cleavage is spotted by the elimination of a CO unit at *m*/*z* 391.

Additionally, neutral losses, which are a projection of the difference between the precursor ion and the fragment ions, are a useful way to detect specific species and confirm MS/MS data.^35^ In this regard, pentacyclic triterpenoids are reported to show such neutral fragments, as described for Sun and co-workers,^33^ where methylene group elimination (−14 Da) followed throughout the fragmentation pattern after the second H_2_O loss, as also seen for compound (**4**) by a fragment ion at *m*/*z* 395.

As described above, the positive mode fragmentation mechanism of pentacyclic triterpenes commonly involves the C-3 substituent bond breakage (e.g., ketone, hydroxyl, or ester group), followed by subsequent eliminations, giving corresponding fragment ions for such compounds.^34^ Therefore, erythrodiol (**5**) [M + H]^+^ *m*/*z* 443 (Table 2 and Figure S7) showed a characteristic initial H_2_O loss from the precursor ion evidenced by a fragment ion at *m*/*z* 425 [M + H–H_2_O]^+^, suggestive of a hydroxyl group at C-3. In addition, an elimination of a methylene group (−14 Da) from the protonated molecule giving a fragment ion at *m*/*z* 411 was also observed, characteristic of (**5**) according to the literature.^34^

Moreover, the ursane-type triterpenoid uvaol (**6**) [M + H]^+^ *m*/*z* 443 was detected in both *E. erythropappus* extracts (LEE and EEE). MS/MS data analysis of (**6**) led to similar attributions of compound (**5**), in addition to the same precursor ion *m*/*z* 443 [M + H]^+^ (Table 2 and Figure S8).^33^ In addition, a fragment ion at *m*/*z* 208, characteristic of a Retro-Diels–Alder rearrangement (RDA), could be detected as being generated from the precursor ion *m*/*z* 443 [M + H]^+^, similar to that reported for other triterpenoid-type compounds.^34^

The friedelane-type ketone friedelin (**7**) was also detected among the annotated pentacyclic terpenoids. In this context, the MS spectrum allowed the detection of a precursor ion at *m*/*z* 427 [M + H]^+^ (Figure S9), related to friedelin in LEE and EEE extracts. MS/MS spectrum was in agreement with attributions made by Huang and co-workers,^36^ where characteristic fragment ions were detected, such as H_2_O elimination from the precursor ion at *m*/*z* 409. In addition, stronger evidence of the presence of this triterpenoid in the extracts was obtained through its further isolation, followed by structural elucidation via NMR spectroscopy.

Lupane-type triterpenoids, unlike previously described compounds, feature a five-membered E ring bonded to an exomethylenic double bond in their pentacyclic core.^37^ Among this class of metabolites, betulinic acid (**8**), known for its activities against *S. mansoni*,^38^ was tentatively annotated in both LEE and EEE extracts (Table 2). The MS/MS spectrum of compound (**8**) [M + H]^+^ *m*/*z* 457 revealed a prominent H_2_O loss from the precursor ion showing a fragment at *m*/*z* 439 [M + H–H_2_O]^+^, indicating a hydroxyl substituent at C-3 consistent with literature findings.^34^ Subsequent alkene formation, similar to oleanane-type isoprenoids (Figure 3), involved a second water loss from the C-28 carboxyl group, resulting in a fragment ion at *m*/*z* 421. Further fragmentation included acylium ion formation and subsequent CO unit elimination (−28 Da), yielding a fragment ion at *m*/*z* 393. Additionally, the elimination of a – HCOOH unit (−46 Da) from *m*/*z* 439 fragment could also be attributed to ion *m*/*z* 393 formation, consistent with observations by Uddin and co-workers^34^ when studying structural–fragmentation relationships of various pentacyclic triterpenes (Figure S10). Additional evidence supporting the annotation of betulinic acid (**8**) as a proposed metabolite was obtained through its isolation and structural elucidation by NMR spectroscopy.

In the negative mode, proposed compounds primarily consisted of flavonoids, such as flavonols, flavones, their glycosides, and phenylpropanoid derivatives (**9–16**) (Table 2).

Kaempferol-7-*O*-neohesperidoside (**12**) [M–H]^−^ *m*/*z* 593, a glycoside derivative commonly found in various plant species,^39^ was spotted in both LEE and EEE extracts. Fragmentation analysis revealed consistent results with those reported by Sánchez-Rabaneda and co-workers,^40^ including sequential losses of deoxyhexose (−146 Da) and hexose (−162 Da) moieties, resulting in fragment ions at *m*/*z* 447 and *m*/*z* 285, respectively (Figure S14). Further detected fragments were consistent with the reported aglycone fragmentation pattern, providing additional evidence for the proposed compound (**12**), which has not been previously reported for *E. erythropappus*.^41^ Furthermore, the 5,7-dihydroxyflavone acacetin (**14**) at *m*/*z* 283 [M–H]^−^ was tentatively annotated through MS/MS spectral analysis, with characteristic fragment ions^42^ indicating methyl group loss at *m*/*z* 268 (Figure S16). Compound (**14**) was also isolated from *E. erythropappus* rinsed extract (LEE), which further corroborates the MS/MS data analysis.

Further investigation into MS/MS fragmentation patterns of di-*O*-caffeoylquinic acids led to the annotation of a di-*O*-caffeoylquinic acid derivative (**16**) [M–H]^−^ *m*/*z* 515 (Figure S18). As far as shown by the literature research, such a metabolite although found in other candeia species (e.g., *Eremanthus crotonoides*)^31^ was not previously reported for *E. erythropappus*. Fragmentation analysis indicated characteristic losses of caffeoyl units (−162 Da), yielding fragment ions at *m*/*z* 353 and *m*/*z* 191,^43^ with additional fragments indicating water loss (−18 Da) at *m*/*z* 335 and decarboxylation (−44 Da) at *m*/*z* 291 from the *m*/*z* 353 fragment ion (Figure S18).

The proposed compounds were consistent with level 2 annotation,^44^ with the negative ionization mode revealing previously unreported metabolites in *E. erythropappus* extracts, including kaempferol-7-*O*-neohesperidoside (**12**) and a di-*O*-caffeoylquinic acid derivative (**16**). On the other hand, UHPLC-ESI-MS/MS analysis in positive mode highlighted distinct chemical profiles between LEE and EEE extracts. Notably, key compounds goyazensolide (**1**), centratherin (**2**), oleanonic acid (**4**), and erythrodiol (**5**) were exclusively detected in LEE. This suggests that the rinsed extraction method enhances the recovery of sesquiterpene lactones and pentacyclic triterpenoids, selectively isolating metabolites from glandular trichomes.

### 3.3 Chemical Identification of Isolated Compounds

Isolated compounds were structurally elucidated through NMR analysis, which followed by literature comparison allowed the identification of the pentacyclic triterpenoids friedelin (**7**) (friedelan-3-one), betulinic acid (3β-hydroxy-20(29)-lupaene-28-oic acid) (**8**), and the flavone acacetin (5,7-dihydroxy-4′-methoxyflavone) (**14**) (Figures S19–S27).

Expansion of the lupane skeleton five-membered ring, followed by several Wagner–Meerwein rearrangements (alkyl and hydride shifts), allowed by an axial–axial conformation of migrating groups, gives friedenyl cation, leading to friedelin upon further oxidation.^45^ Friedelin (**7**) (Figure 3), previously reported for *E. erythropappus*,^46,47^ was isolated in this work, showing similar attributions to the reported literature,^48^ allowing the identification of this metabolite (Figures S21 and S24). ^1^H NMR (500 MHz, CDCl_3_) δ (ppm): δ 2.37 (1H, ddd), 2.28 (2H, dq), 1.95 (1H, m), 1.75 (1H, d), 1.70 (1H, qd), 1.60 (1H, m), 1.55 (2H, m), 1.45 (3H, m), 1.38 (3H, m), 1.26 (3H, m), 1.18 (3H, s), 1.05 (3H, s), 1.00 (6H, d), 0.95 (3H, s), 0.87 (6H, d), 0.73 (3H, s). ^13^C NMR (125 MHz, CDCl_3_) δ (ppm): δ 213.3, 59.5, 58.2, 53.1, 42.8, 42.2, 41.5, 41.3, 39.7, 39.3, 38.3, 37.5, 36.0, 35.6, 35.4, 35.0, 32.8, 32.4, 32.1, 31.8, 30.5, 30.0, 28.2, 22.3, 20.3, 18.7, 18.2, 18.0, 14.7, 6.8 (Figures S19 and S20).

Characteristic chemical shifts were attributed to betulinic acid (**8**) ^1^H NMR (500 MHz, DMSO-*d*_6_) δ (ppm): 12.03 (1H, s), 4.68 (1H, s), 4.56 (1H, s), 4.24 (1H, d), 2.23 (2H, m), 2.22 (1H, td), 2.10 (1H, d), 1.80 (2H, d), 1.64 (3H, s), 1.55–1.07 (23H, m), 0.93 (3H, s), 0.87 (6H, s), 0.76 (3H, s), 0.65 (3H, s), ^13^C NMR (125 MHz, DMSO-*d*_6_) δ (ppm): 177.1, 150.2, 109.5, 76.7, 55.3, 54.8, 49.9, 48.5, 46.5, 41.9, 40.2, 38.4, 38.2, 37.5, 36.7, 36.3, 33.9, 31.6, 30.1, 29.1, 28.1, 27.1, 25.0, 20.4, 18.9, 17.9, 15.9, 15.7, 15.7, 14.3. In this context, a singlet (s) at 12.03 ppm was readily spotted, being related to a deshielded carboxylic acid hydrogen. Moreover, the allylic exomethylene double bond was observed as two singlets (4.68 1H, s 4.56 1H, s) related to weak coupling diastereotopic hydrogens, followed by a broad doublet (d) of the hydroxyl hydrogen at 4.24 ppm (1H, d) coupled in a vicinal manner (*J*_3_ = 5 Hz) with the C-3 carbinolic hydrogen (2.22, 1H, td) (Figure S21), which was better confirmed after conducting an additional ^1^H NMR experiment by adding D_2_O into the NMR tube, leading to 80% of acidic hydroxyl hydrogen exchange with deuterium and disappearance of the carboxylic acid hydrogen (Figures S22 and S23). A characteristic methyl group pattern of the lupane scaffold throughout the biosynthetic pathway^37^ was established due to signals ^1^H NMR observed as singlets in 1.64 (3H, s), 0.93 (3H, s), 0.87 (6H, s), 0.76 (3H, s), 0.65 (3H, s) chemical shifts, suggestive of noncoupling homotopic hydrogens of six methyl groups (Figure S21). ^13^C NMR showed similar attributions of those reported from Pohjala and co-workers,^49^ which in addition to the carboxylic acid carbonyl group (177.3 ppm), allylic exomethylene double bond (150.2 and 109.5 ppm), and C-3 bonded to hydroxyl group (76.7 ppm). A distinguishable signal among other lupanoids (e.g., lupeol, betulin) was brought by a 55.3 ppm chemical shift, suggestive of C-17 as sp^3^ carbon atom further deshielded due to bonding with an electron-withdrawing carboxyl group (Figure S24). In addition, the hydrogenated carbon pattern, consisting of 6 methyl, 6 methines, and 11 methylene groups, was assigned in the DEPT-135 NMR (Figure S25), leading to identification of the pentacyclic triterpenoid betulinic acid (**8**) (Figure 3) for the first time isolated from *E. erythropappus*.

Another class of metabolites often reported for *Eremanthus* species is flavonoids.^50^ In this context, the flavone acacetin (**14**) (Figure 3) was also isolated from this plant. ^1^H NMR (500 MHz, DMSO-*d*_6_) δ (ppm): 12.90 (1H, s), 8.02 (2H, d), 7.10 (2H, d), 6.84 (1H, s), 6.48 (1H, s), 6.18 (1H, s), 3.85 (3H, s), ^13^C NMR (125 MHz, DMSO-*d*_6_) δ (ppm): 182.2, 165.2, 163.7, 162.8, 161.9, 157.9, 128.8, 123.4, 115.1, 104.1, 104.0, 99.5, 94.6, 56.0 (Figures S26 and S27). These metabolites commonly feature a low-field C-5 hydroxyl proton (5-OH) of the A-ring due to hydrogen bonding with the C-ring carbonyl group, such as hydroxylated flavones reported by Aksnes and co-workers.^51^ Therefore, ^1^H NMR analysis of compound (**14**) presented such a characteristic signal at 12.92 ppm (1H, s), suggestive of the 5-OH hydrogen deshielded by ketone interaction (182.2 ppm).^52^ Subsequent attributions at the spectrum lead to knowledge of the substitution pattern at both A and B rings; as such, doublets at 8.02 (2H, d) and 7.10 (2H, d) chemical shifts are suggestive of a plane of symmetry in a disubstituted pattern in the B-ring due to the presence of a C-4′methoxy group evidenced by noncoupling homotopic hydrogens at 3.85 (3H, s), resulting in two *ortho* couplings (*J*_3_ = 10 Hz) on each side of the B-ring (Figure S26). Additional evidence of a 5, 7-hydroxyflavone isolation was gathered by ^13^C NMR (Figure S27), where characteristic C-6 and C-8 in the A-ring appeared at 99.5 and 94.6 ppm, respectively, in which such a shielding may be attributed to *ortho*- and *para*-directing groups at positions C-5 (162.8 ppm) and C-7 (165.2 ppm), similar to chrysin, another 5, 7-hydroxyflavone.^53^

### 3.4 *In Vitro* Antischistosomal Activity of Isolated Compounds from LEE and Cytotoxicity Assessment

Among the isolated compounds, betulinic acid (**8**) emerged as the primary active constituent, exhibiting significant antischistosomal activity (EC_50_ = 36.8 μM) (Figure 4A,B), high selectivity (SI > 15.5), and no cytotoxicity (CC_50_ > 500 μM) (Table 1). In contrast, friedelin (**7**) and acacetin (**14**) were inactive at concentrations of up to 50 μM (Table 1). While previous studies reported limited efficacy for betulinic acid (**8**) against *S. mansoni*,^38^ the observed discrepancies may stem from differences in experimental conditions, such as solubility or exposure time.^54^ The efficacy demonstrated by betulinic acid (**8**) in this study underscores its promise as a lead molecule and supports the need for additional research into its mechanism of action. Although we have not yet evaluated combinations of isolated metabolites, the markedly greater efficacy of LEE compared to that of the isolated betulinic acid suggests the potential for synergistic interactions among its constituents, warranting further investigation.^55^

**Figure 4:**
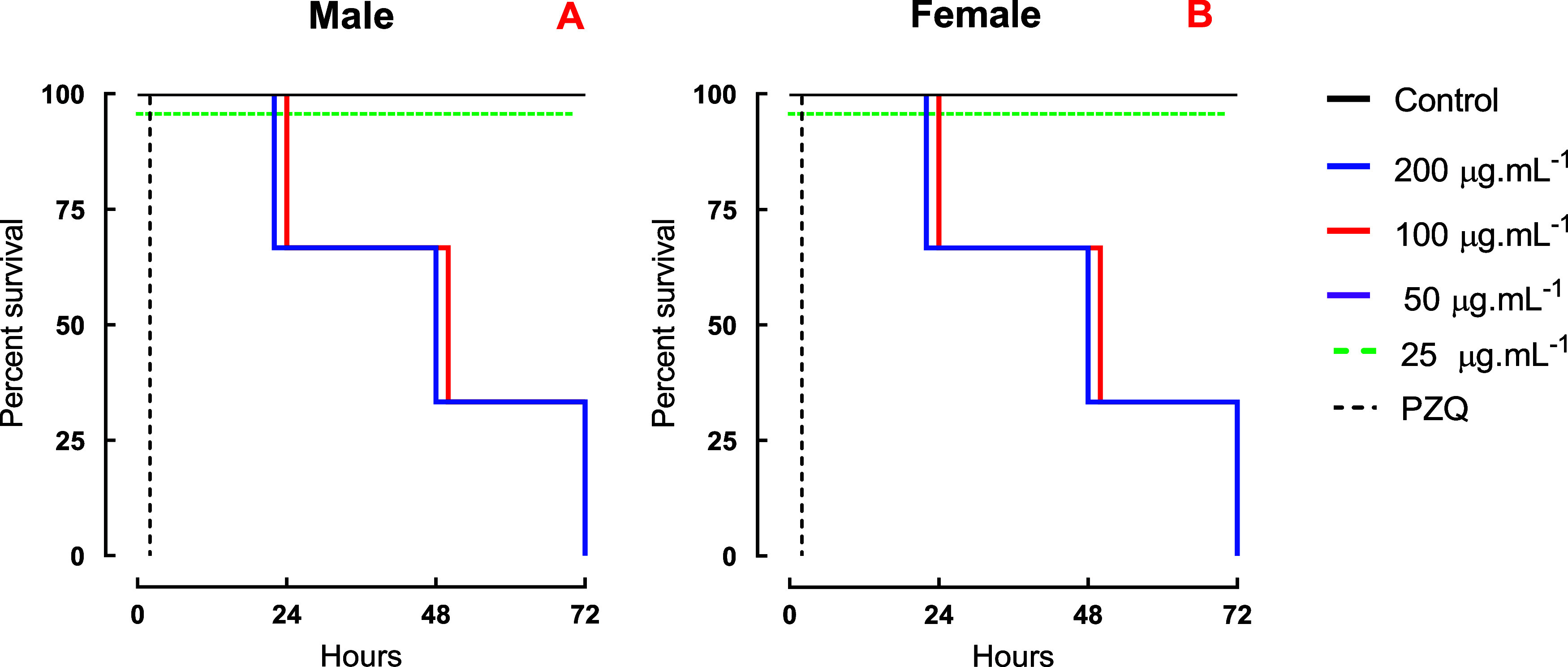
Schistosomicidal effects of betulinic acid on *S. mansoni* adult worms were assessed *in vitro*. Male (A) and female (B) parasites were observed for a duration of 72 h, and the outcomes are presented as the percentage of survival based on Kaplan–Meier survival curves. The average viability values were calculated from a minimum of three separate experiments (*n* = 3). Control: 0.5% DMSO in RPMI 1640 medium. PZQ: 2 μM (0.62 μg/mL).

### 3.5 *In Vivo* Efficacy of LEE Extract and Betulinic Acid (**8**) in *S. mansoni*-Infected Mice

After 49 days of infection in mice, treatment with LEE (400 mg/kg) resulted in a significant reduction of worm burden by 86.2% (*P* < 0.01), while betulinic acid (**8**) (400 mg/kg) achieved a 41.9% reduction (*P* < 0.01). Both treatments were compared to the control group, and PZQ demonstrated a remarkable reduction of 92.6% in worm burden (*P* < 0.0001) (Figure 5). Previous studies have indicated that betulinic acid (**8**) exhibited limited efficacy *in vitro*.^38^ However, to the best of our knowledge, no research has been published regarding the *in vivo* antischistosomal efficacy.

**Figure 5:**
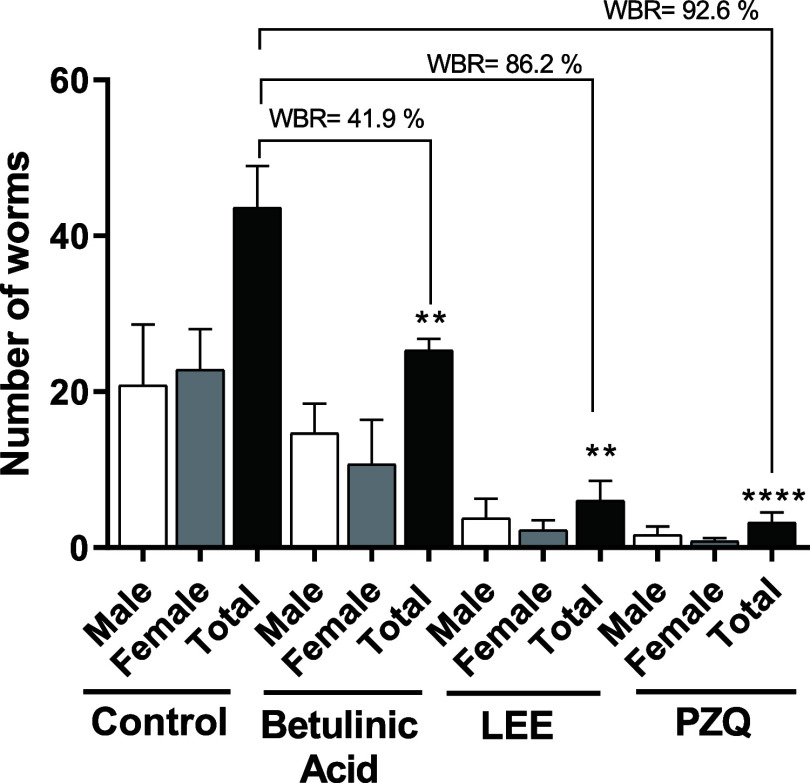
Impact of a single dose of betulinic acid (400 mg/kg, p.o.), LEE (400 mg/kg, p.o.), and PZQ (400 mg/kg, p.o.) on worm burden in mice with a 49 day old adult *S. mansoni* infection, separated by gender, is depicted. The bars represent data from individual mice that were either infected and treated with betulinic acid, LEE, and PZQ or infected and treated with the control (vehicle). **P < 0.01, ****P < 0.0001 compared to the control groups. WBR, worm burden reduction.

Oral administration of the LEE extract (400 mg/kg) also resulted in a significant reduction of 86.9% in immature egg deposition and a 33.3% inhibition of mature egg production (Figure 6A). Fecal analysis of treated mice revealed an 80.9% (*P* < 0.05) reduction in the number of eggs per gram compared to the control group (Figure 6B). This is particularly noteworthy, as egg deposition is a key factor in the pathogenesis and transmission of schistosomiasis.^56^ Compared to previous studies on other plant extracts, such as the extracts from *A. oleracea* ^28^ and *P. umbellata*,^21^ which showed 32.6 and 60% reductions, respectively, the LEE extract shows a notably higher potential for reducing egg deposition. These results suggest that LEE could be a promising lead for future therapeutic development.

**Figure 6:**
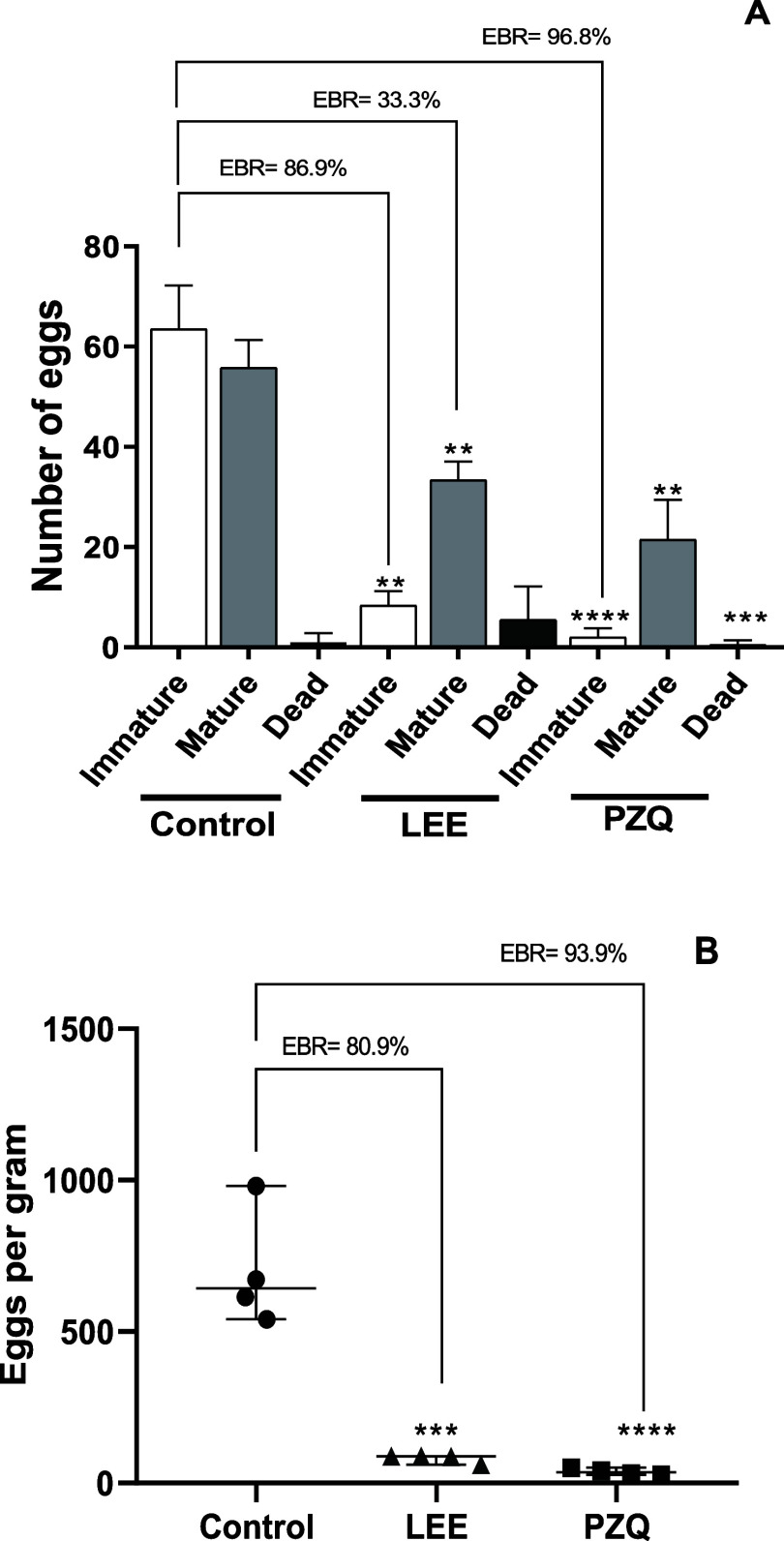
Effects on egg development stages (oogram) (A) and egg load (B) of LEE and PZQ administered (400 mg/kg, p.o.) to mice harboring a 49 day old adult *S. mansoni* infection. Bars and points represent data from individual mice that were infected and treated with samples or infected and treated with vehicle (control). **P < 0.01, ***P < 0.001, and ****P < 0.0001 compared with control groups. EBR: egg burden reduction.

Overall, our findings highlight the significant *in vivo* antischistosomal effects of both LEE and betulinic acid (**8**), with LEE demonstrating markedly greater efficacy. While **8** may contribute to the observed activity, the enhanced effects of LEE may be likely due to the synergistic action of other compounds in the extract, such as STLs and triterpenoids.

Our findings suggest that the *in vivo* antischistosomal effects of betulinic acid (**8**) may be modulated by various factors, such as its bioavailability, metabolic processing in the host, and potential interactions with the immune system, which could collectively enhance its efficacy against *S. mansoni*. However, these aspects require a more detailed exploration. Although the exact mechanism remains to be elucidated, we hypothesize that betulinic acid may interfere with mitochondrial function or redox balance,^57^ as previously reported for other pentacyclic triterpenoids in helminths and protozoan parasites.^58,59^ Moreover, betulinic acid and related triterpenoids are known to modulate various cellular pathways and signaling molecules, which could contribute to their antiparasitic effects.^60,61^ This underlines the necessity of further research to unravel the precise mechanisms underlying the antischistosomal activity of betulinic acid (**8**) in the murine *S. mansoni* infection model. Also, the superior efficacy of LEE over the isolated compound emphasizes the potential advantages of whole-plant extracts, which may offer a more comprehensive therapeutic strategy against schistosomiasis.

## 4 Conclusions

In conclusion, the results of this study highlight the promising antischistosomal potential of *E. erythropappus* extracts, with the rinsed extract (LEE) demonstrating greater activity compared to that of the ethanolic extract (EEE) against *S. mansoni*. UHPLC-ESI-MS/MS analysis of both LEE and EEE extracts annotated a total of 16 compounds, including sesquiterpene lactones and pentacyclic triterpenoids as key differences among the extracts. From the LEE extract, the compounds friedelin (**7**), betulinic acid (**8**), and acacetin (**14**) were isolated, with betulinic acid (**8**) and acacetin (**14**) being reported here for the first time in this species. While friedelin (**7**) and acacetin (**14**) exhibited no activity, betulinic acid (**8**) demonstrated significant *in vitro* and *in vivo* antischistosomal effects without causing toxicity to mammalian cells. Additionally, LEE treatment significantly reduced the worm burden and egg production *in vivo*. These findings also emphasize the potential of LEE and its isolated compounds in the development of new natural products for the treatment of schistosomiasis and underline the importance of optimizing extraction methods to isolate bioactive compounds.

## Supplementary Material



## Notes

UHPLC-ESI-MS from extracts and MS/MS spectra of annotated compounds from *E. erythropappus* aerial part extracts (LEE and EEE) and NMR spectra of isolated compounds **7**, **8**, and **14** (PDF)
