# Supplementary material for: Eremanthus erythropappus as a Source of Antiparasitic Agents: In Vitro and In Vivo Efficacy against Schistosoma mansoni
Source: ACS Omega. 2025 Jul 9;10(28):30773–84. doi: 10.1021/acsomega.5c03005 (PMC12290960; doi:10.1021/acsomega.5c03005)

Supporting Information

Eremanthus erythropappus as a source of antiparasitic agents: *in vitro* and *in vivo* efficacy against *Schistosoma mansoni*

Leonardo Luiz O. de Mello [a,b], Carla M. Leal [a] , Monique C. Amaro[c], Allan I. Andrade-de-Siqueira [c], Josué de Moraes [c,d], Ademar A. da Silva Filho [a*], Giovanni W. Amarante [b*]

[a]Núcleo de Identificação e Pesquisa em Princípios Ativos Naturais (NIPPAN), Faculdade de Farmácia, Departamento de Ciências Farmacêuticas, Universidade Federal de Juiz de Fora, R. José Lourenço Kelmer s/n, Campus Universitário, Juiz de Fora, MG, 36036-900, Brazil

[b]Grupo de Pesquisas em Metodologias Sintéticas (GPMS), Departamento de Química, Instituto de Ciências Exatas, Universidade Federal de Juiz de Fora, R. José Lourenço Kelmer s/n, Campus Universitário, Juiz de Fora, MG, 36036-900, Brazil

[c]Núcleo de Pesquisa em Doenças Negligenciadas (NPDN), Universidade Guarulhos, Guarulhos, SP, 07023-070, Brazil

[d]Núcleo de Pesquisa em Doenças Negligenciadas, Instituto Científico e Tecnológico, Universidade Brasil, São Paulo, SP, 08230-030, Brazil.

Corresponding authors

Giovanni Wilson Amarante- *Grupo de Pesquisas em Metodologias Sintéticas (GPMS), Departamento de Química, Instituto de Ciências Exatas, Universidade Federal de Juiz de Fora, R. José Lourenço Kelmer s/n, Campus Universitário, Juiz de Fora, MG, 36036-900, Brazil; E-mail:* giovanni.amarante@ufjf.br

Ademar Alves da Silva Filho- *Núcleo de Identificação e Pesquisa em Princípios Ativos Naturais (NIPPAN), Faculdade de Farmácia, Departamento de Ciências Farmacêuticas, Universidade Federal de Juiz de Fora, R. José Lourenço Kelmer s/n, Campus Universitário, Juiz de Fora, MG, 36036-900, Brazil*; Email: ademar.alves@ufjf.br

Table of contents **Page No.**

S1 UHPLC-ESI-MS/MS *Eremanthus erythropappus* LEE (A) and EEE (B) Extracts in positive mode..**S4**

S2 UHPLC-ESI-MS/MS *Eremanthus erythropappus* LEE (A) and EEE (B) Extracts in negative mode **S4**

S3 Goyazensolide MS/MS spectrum *m/z* 361 $[M+H]^+$..**S5**

S4 Centhraterin MS/MS spectrum *m/z* 375 $[M+H]^+$..**S5**

S5 Lychnopholide MS/MS spectrum *m/z* 359 $[M+H]^+$ LEE and EEE extracts..........................**S6**

S6 Oleanonic acid MS/MS spectrum *m/z* 455 $[M+H]^+$..**S7**

S7 Erythrodiol MS/MS spectrum *m/z* 443 $[M+H]^+$..**S7**

S8 Uvaol MS/MS spectrum *m/z* 443 $[M+H]^+$ LEE and EEE extracts..**S8**

S9 Friedelin MS/MS spectrum *m/z* 427 $[M+H]^+$ LEE and EEE extracts..**S9**

S10 Betulinic acid MS/MS spectrum *m/z* 457 $[M+H]^+$ LEE and EEE extracts..........................**S10**

S11 Apigenin MS/MS spectrum *m/z* 269 $[M-H]^-$ LEE and EEE extracts..........................**S11**

S12 Isoquercetin MS/MS spectrum *m/z* 463 $[M-H]^-$ EEE extract..**S12**

S13 Isorhamnetin-3-*O*-galactoside MS/MS spectrum *m/z* 477 $[M-H]^-$ EEE extract................**S13**

S14 Kaempferol-7-*O*-neohesperidoside MS/MS spectrum *m/z* 593 $[M-H]^-$ LEE and EEE extracts..**S14**

S15 Vicenin-II MS/MS spectrum *m/z* 593 $[M-H]^-$ EEE extract..**S15**

S16 Acacetin MS/MS spectrum *m/z* 283 $[M-H]^-$ LEE and EEE extracts..........................**S16**

S17 Luteolin MS/MS spectrum *m/z* 285 $[M-H]^-$ LEE and EEE extracts..........................**S17**

S18 Di-*O*-caffeoylquinic acid MS/MS spectrum *m/z* 515 $[M-H]^-$ LEE and EEE extracts..**S18**

S19 ^1H NMR spectrum of friedelin in $CDCl_3$..**S19**

S20 ^{13}C NMR spectrum of friedelin in $CDCl_3$..**S20**

S21 ^1H NMR spectrum of betulinic acid in DMSO-d_6..**S21**

S22 ^{1}H NMR spectrum of betulinic acid with added D_2O..**S22**

S23 Betulinic acid hydroxyl hydrogen exchange with D_2O solvent.......................................**S23**

S24 ^{13}C NMR spectrum of betulinic acid in DMSO-d_6……………………….......................**S24**

S25 DEPT-135 NMR spectrum of betulinic acid in DMSO-d_6…………….......................**S25**

S26 ^{1}H NMR spectrum of acacetin in DMSO-d_6……………………………..........................**S26**

S27 ^{13}C NMR spectrum of acacetin in DMSO-d_6…………………………............................**S27**

Figure S1. UHPLC-ESI-MS/MS *Eremanthus erythropappus* LEE (A) and EEE (B) Extracts in positive mode

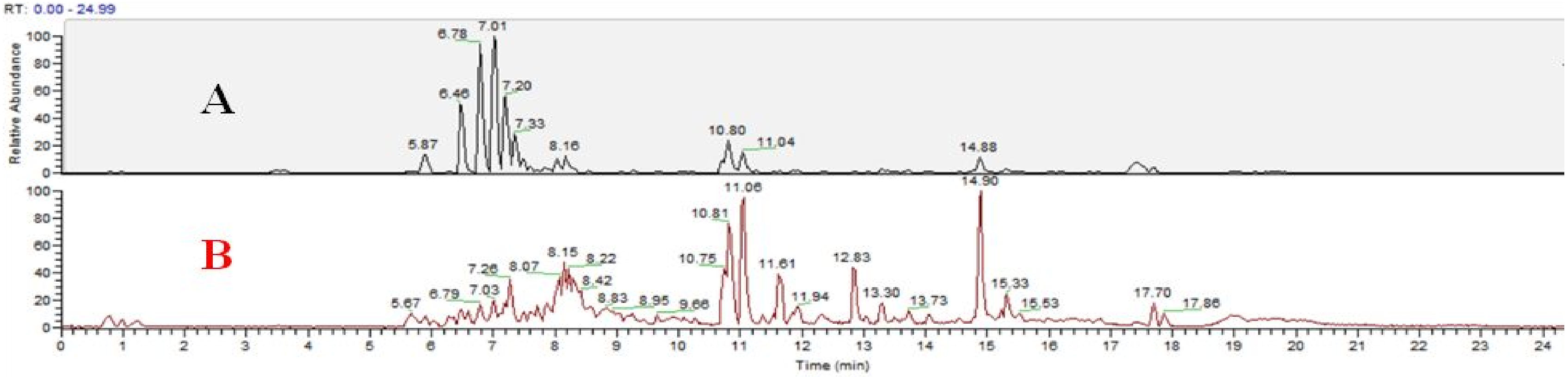

Figure S2. UHPLC-ESI-MS/MS *Eremanthus erythropappus* LEE (A) and EEE (B) Extracts in negative mode

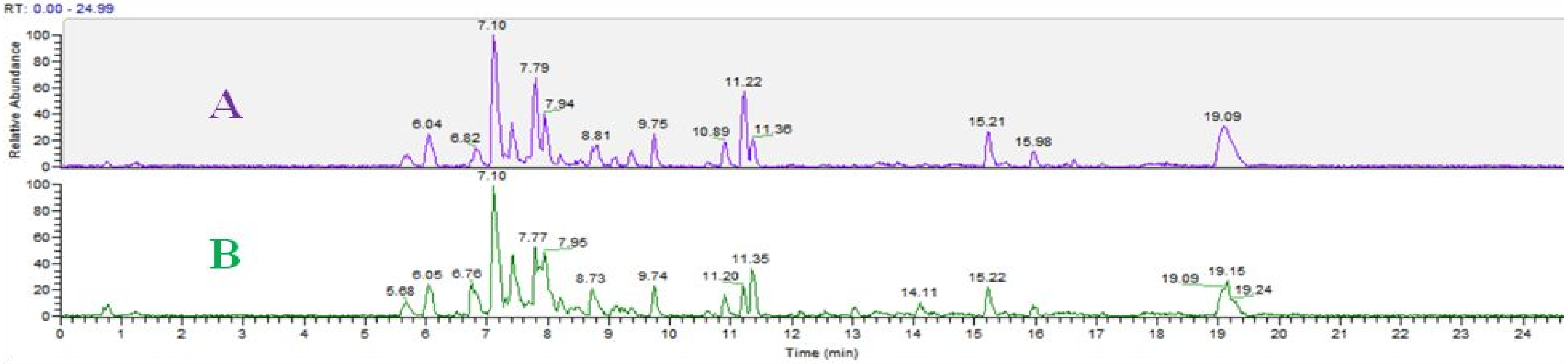

Figure S3. Goyazensolide MS/MS spectrum *m/z* 361 $[M+H]^+$

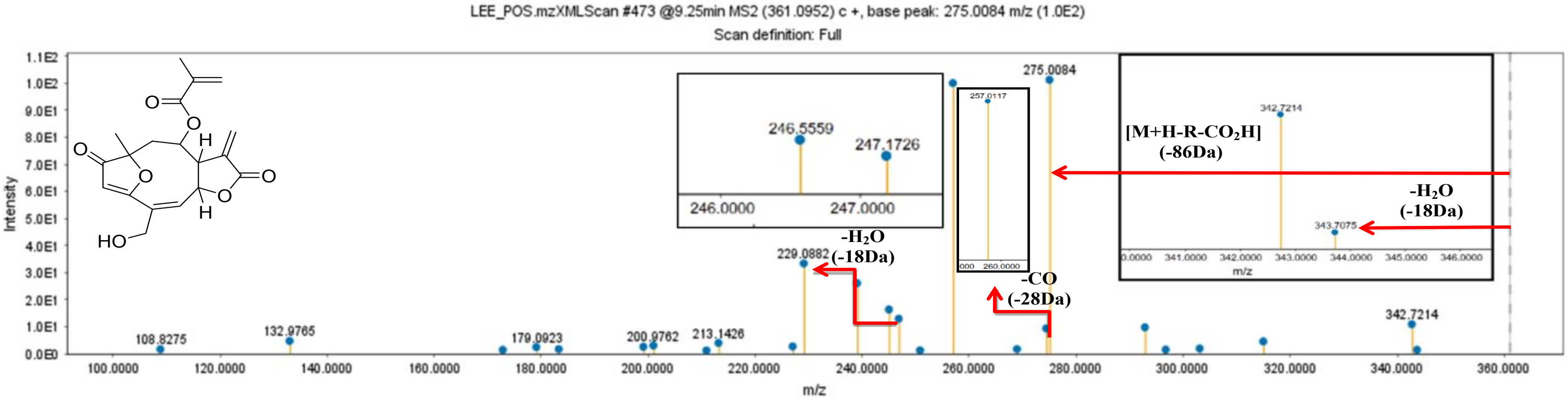

Figure S4. Centhraterin MS/MS spectrum *m/z* 375 $[M+H]^+$

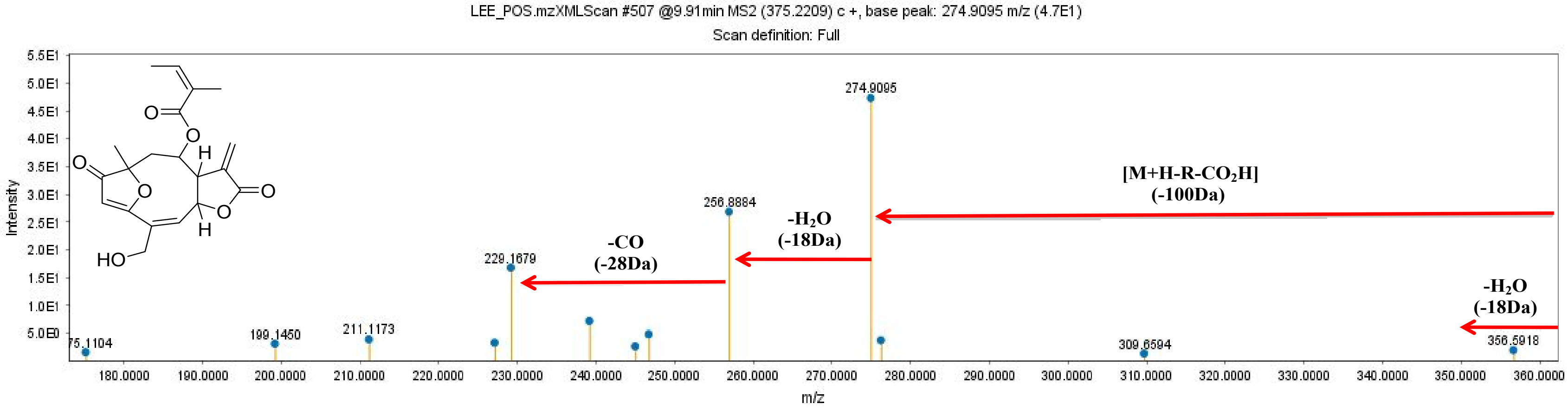

Figure S5. Lychnopholide MS/MS spectrum *m/z* 359 $[M+H]^+$ LEE and EEE extracts

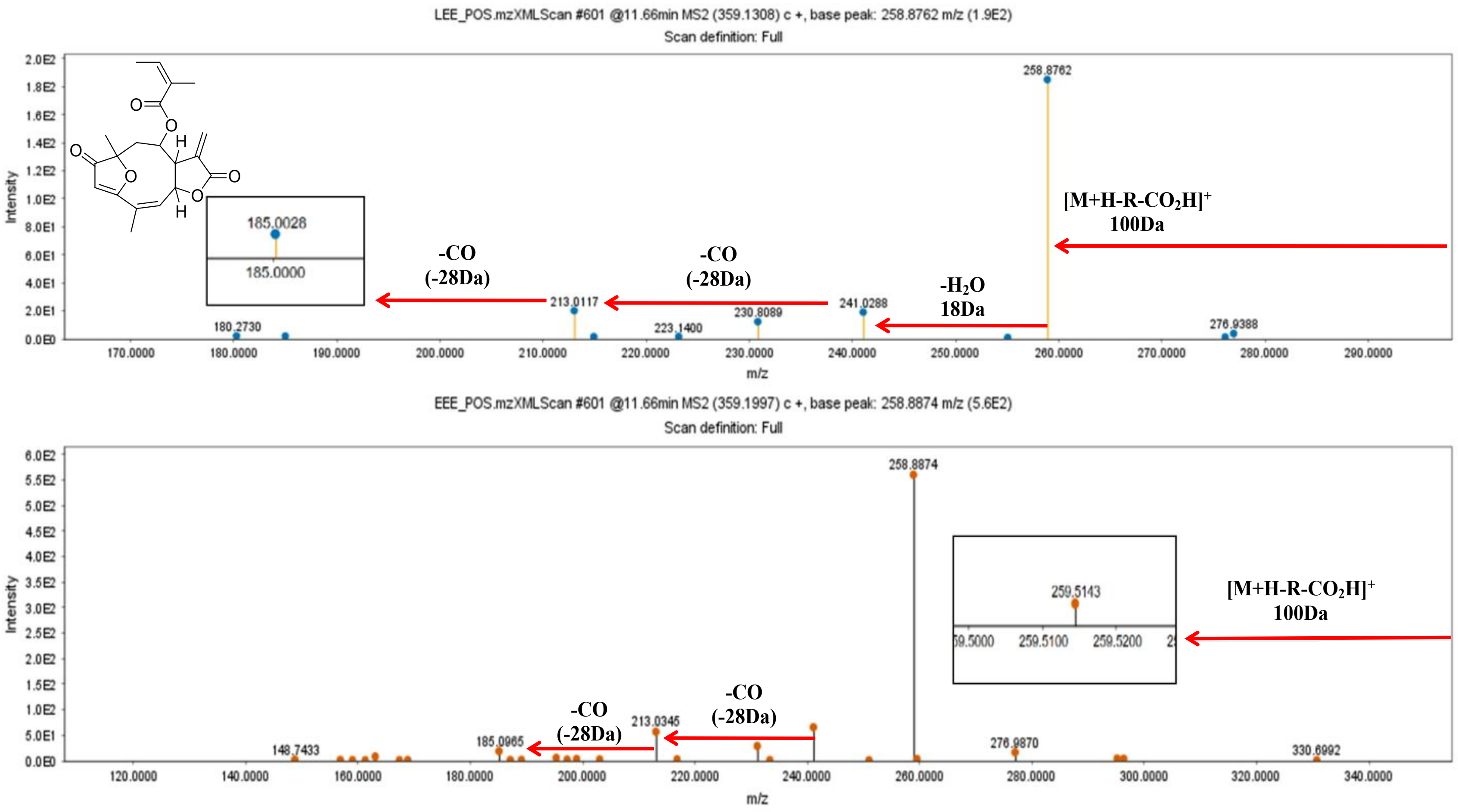

Figure S6. Oleanonic acid MS/MS spectrum *m/z* 455 [M+H]$^+$

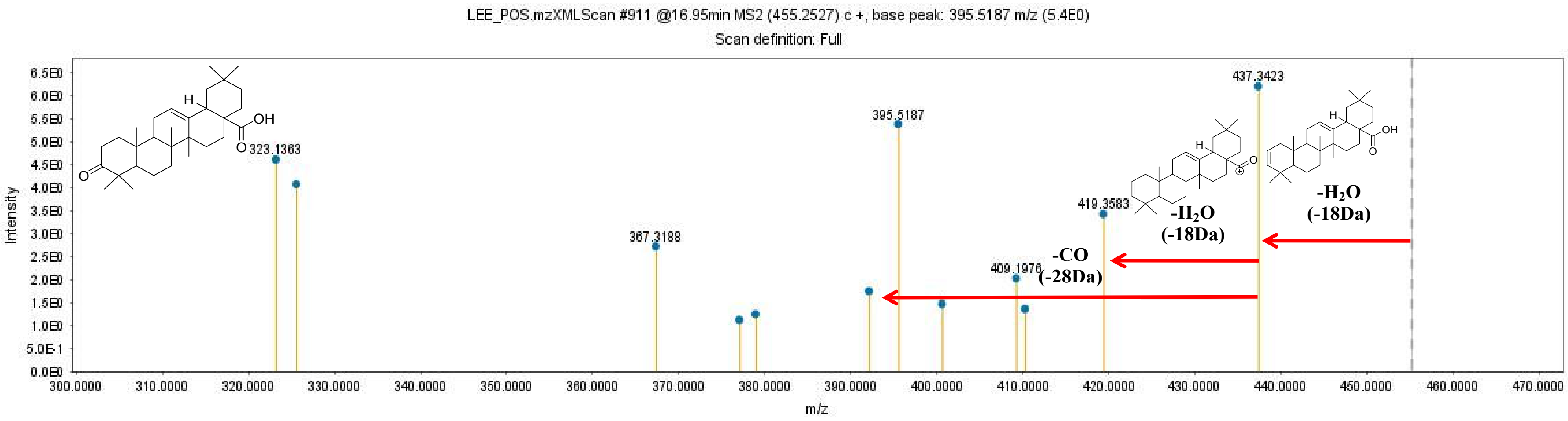

Figure S7. Erythrodiol MS/MS spectrum *m/z* 443 [M+H]$^+$

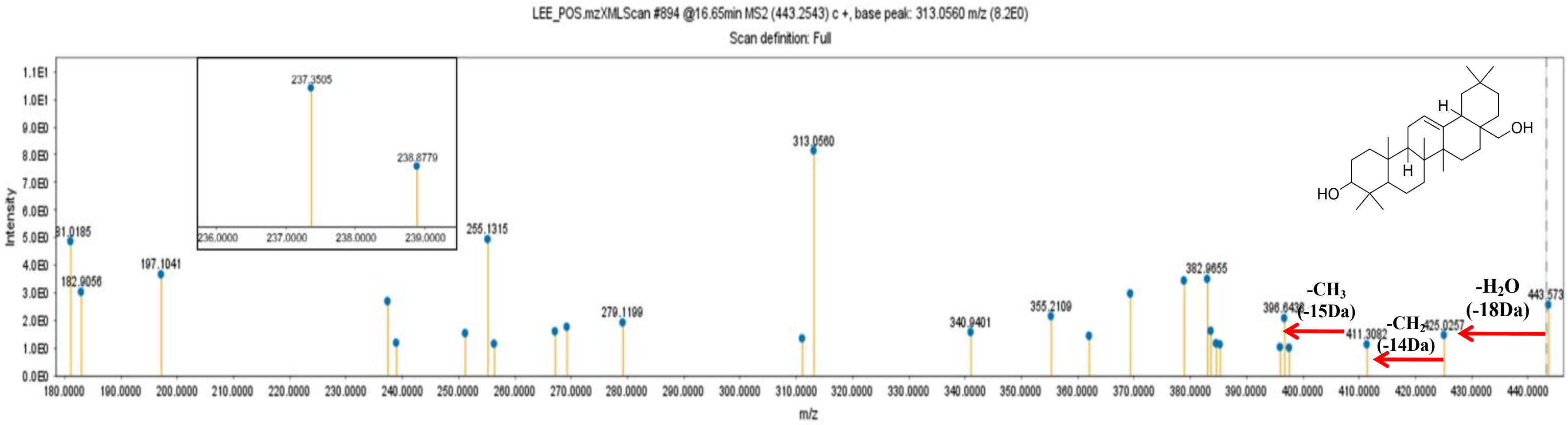

Figure S8. Uvaol MS/MS spectrum *m/z* 443 $[M+H]^+$ LEE and EEE extracts

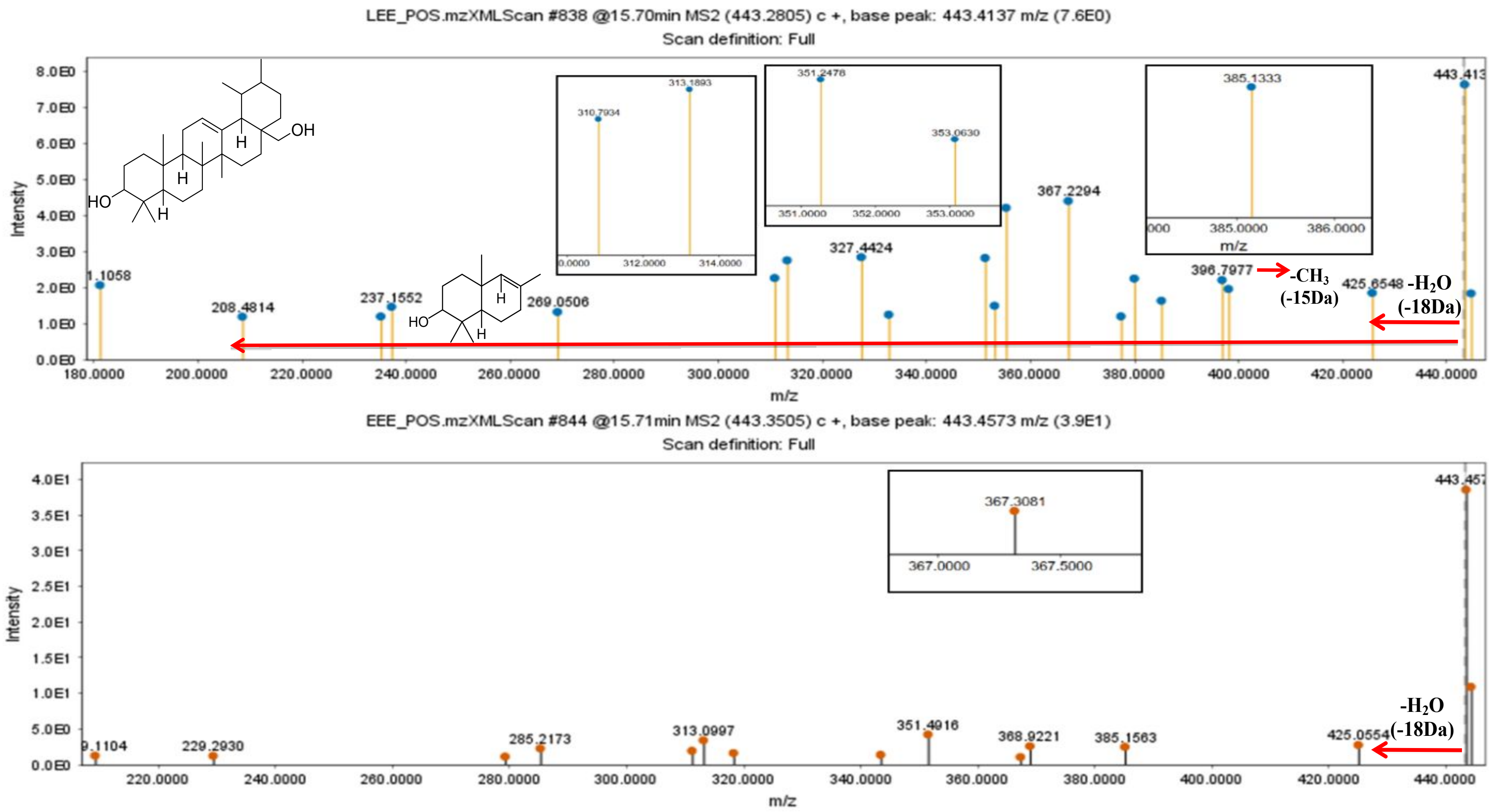

Figure S9. Friedelin MS/MS spectrum *m/z* 427 $[M+H]^+$ LEE and EEE extracts

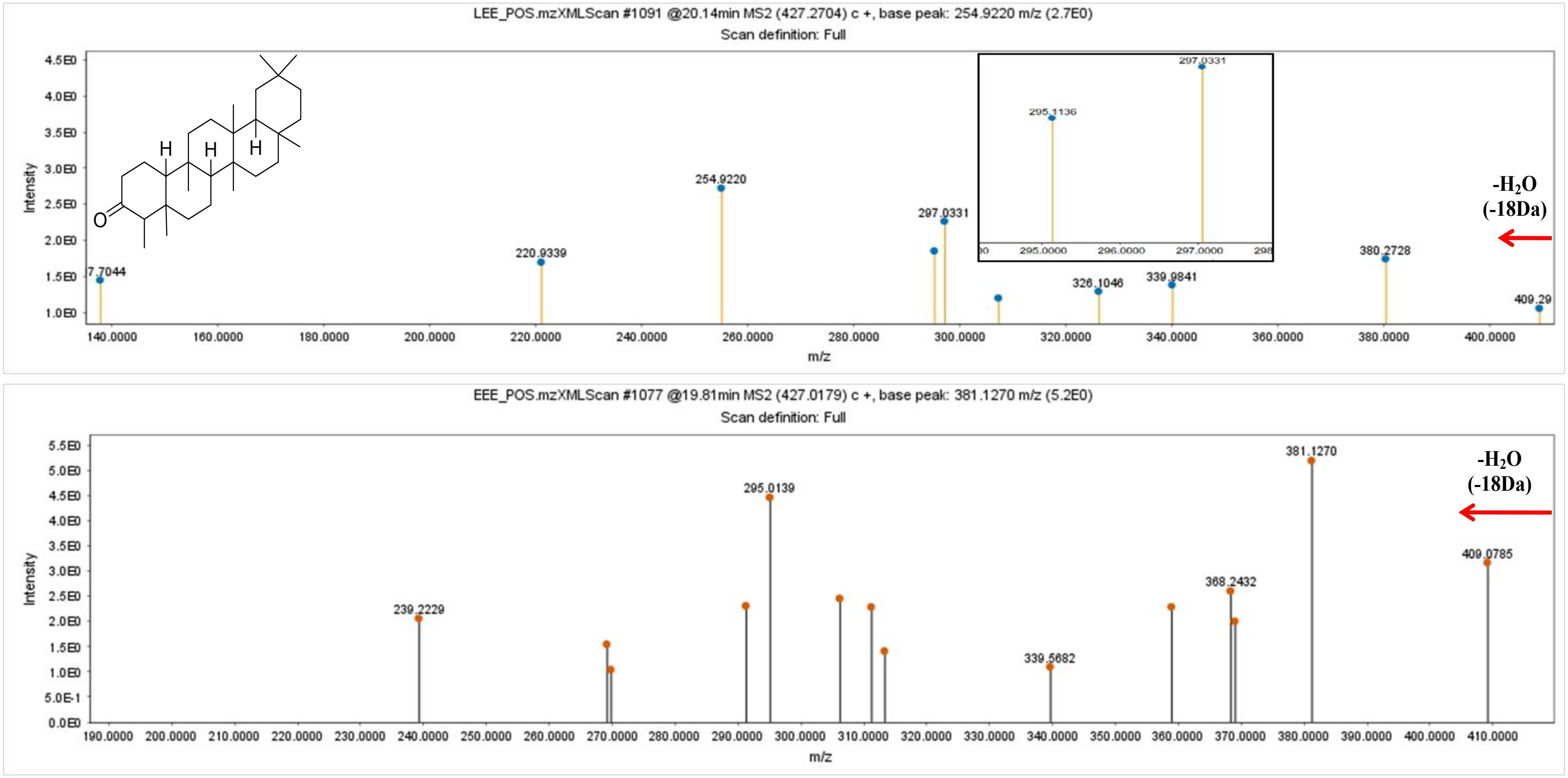

Figure S10. Betulinic acid MS/MS spectrum *m/z* 457 $[M+H]^+$ LEE and EEE extracts

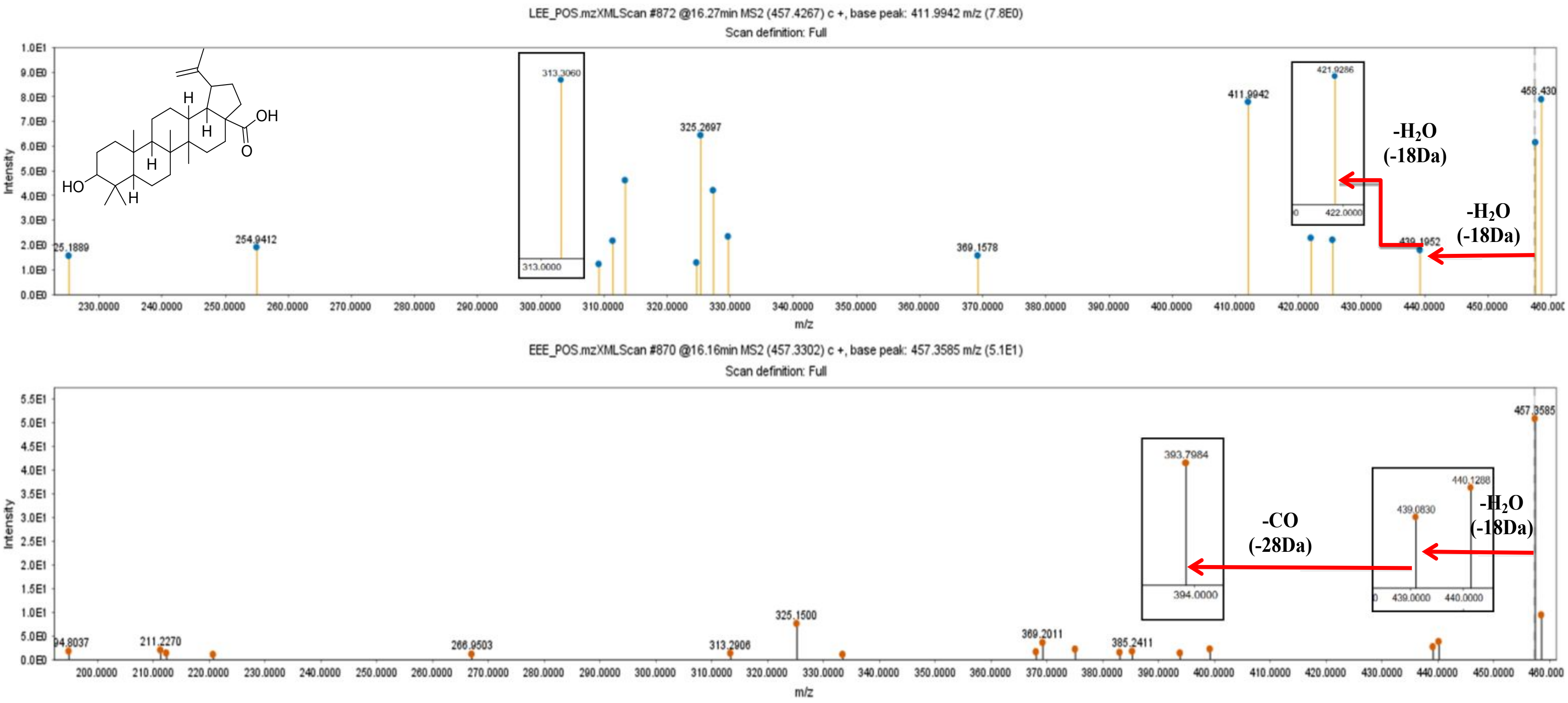

Figure S11. Apigenin MS/MS spectrum *m/z* 269 [M-H]$^-$ LEE and EEE extracts

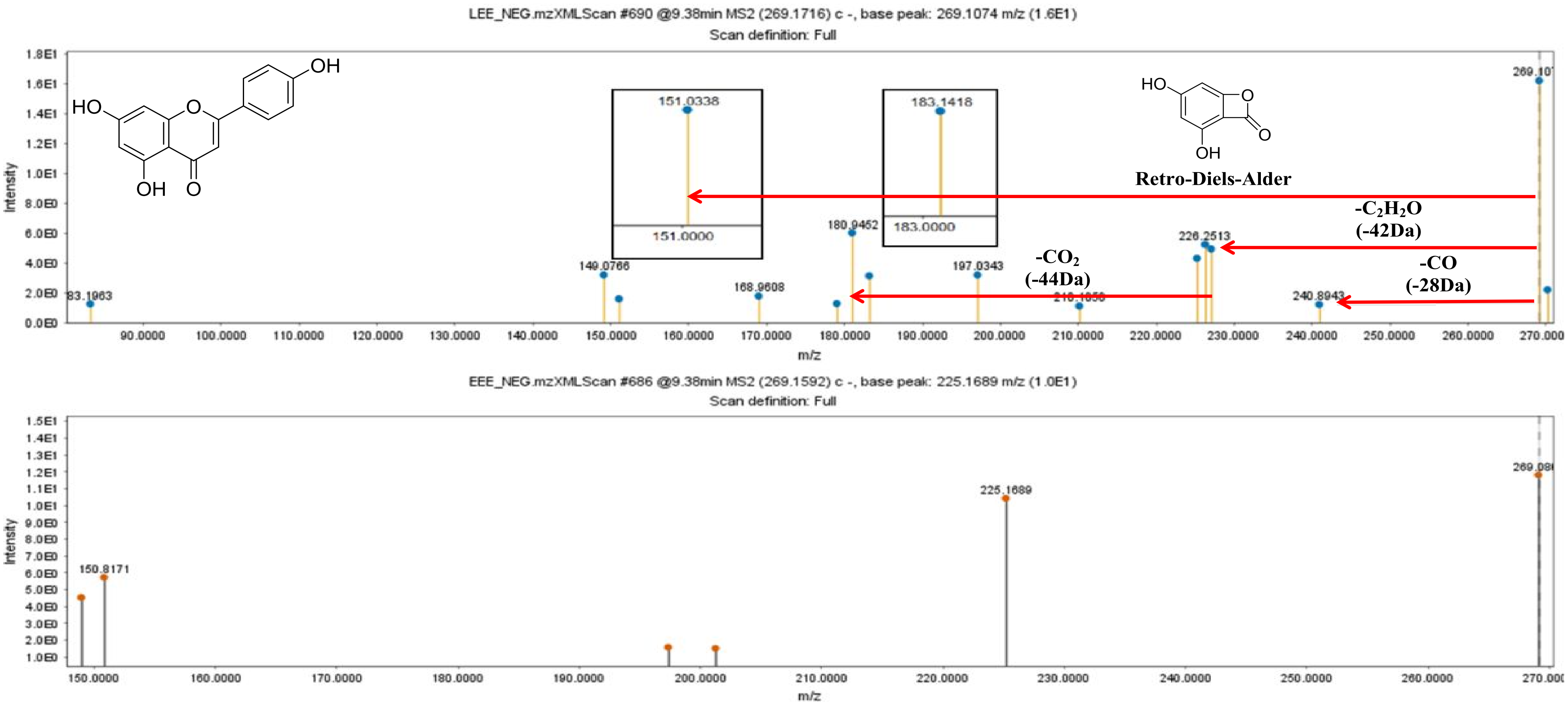

Figure S12. Isoquercetin MS/MS spectrum *m/z* 463 [M-H]$^-$ EEE extract

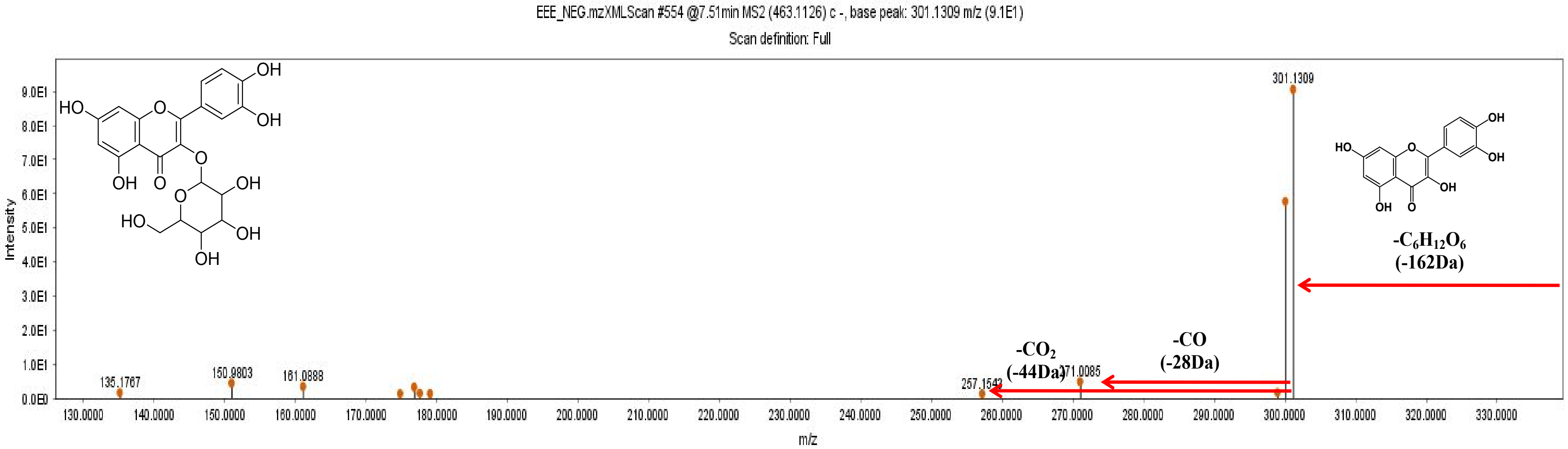

Figure S13. Isorhamnetin-3-*O*-galactoside MS/MS spectrum *m/z* 477 [M-H]$^-$ EEE extract

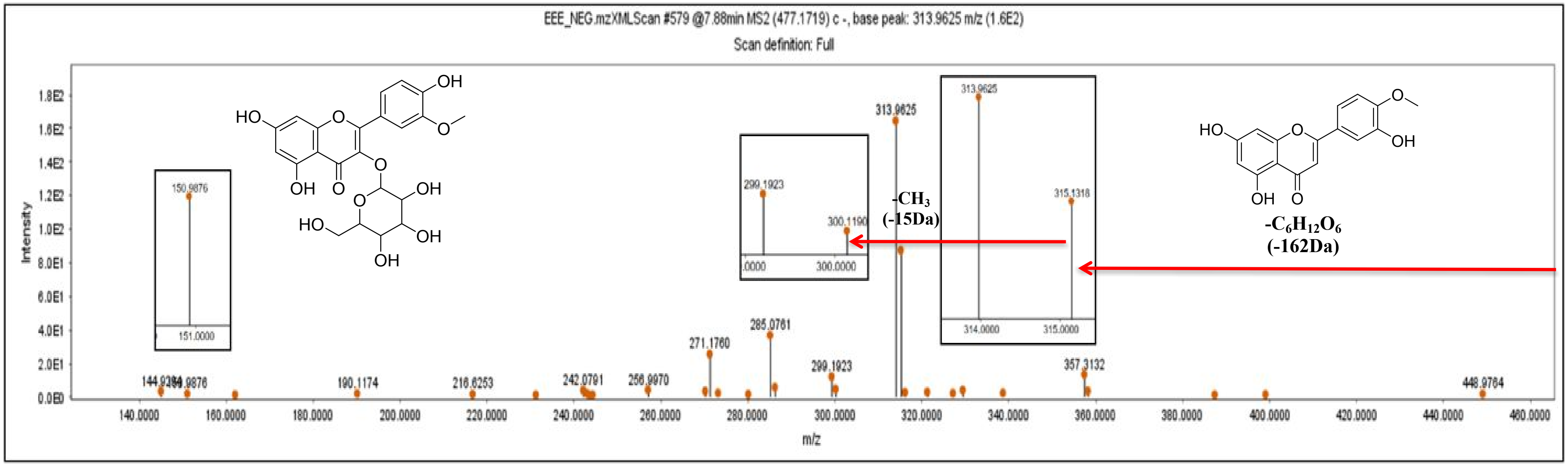

Figure S14. Kaempferol-7-*O*-neohesperidoside MS/MS spectrum *m/z* 593 $[M-H]^-$ LEE and EEE extracts

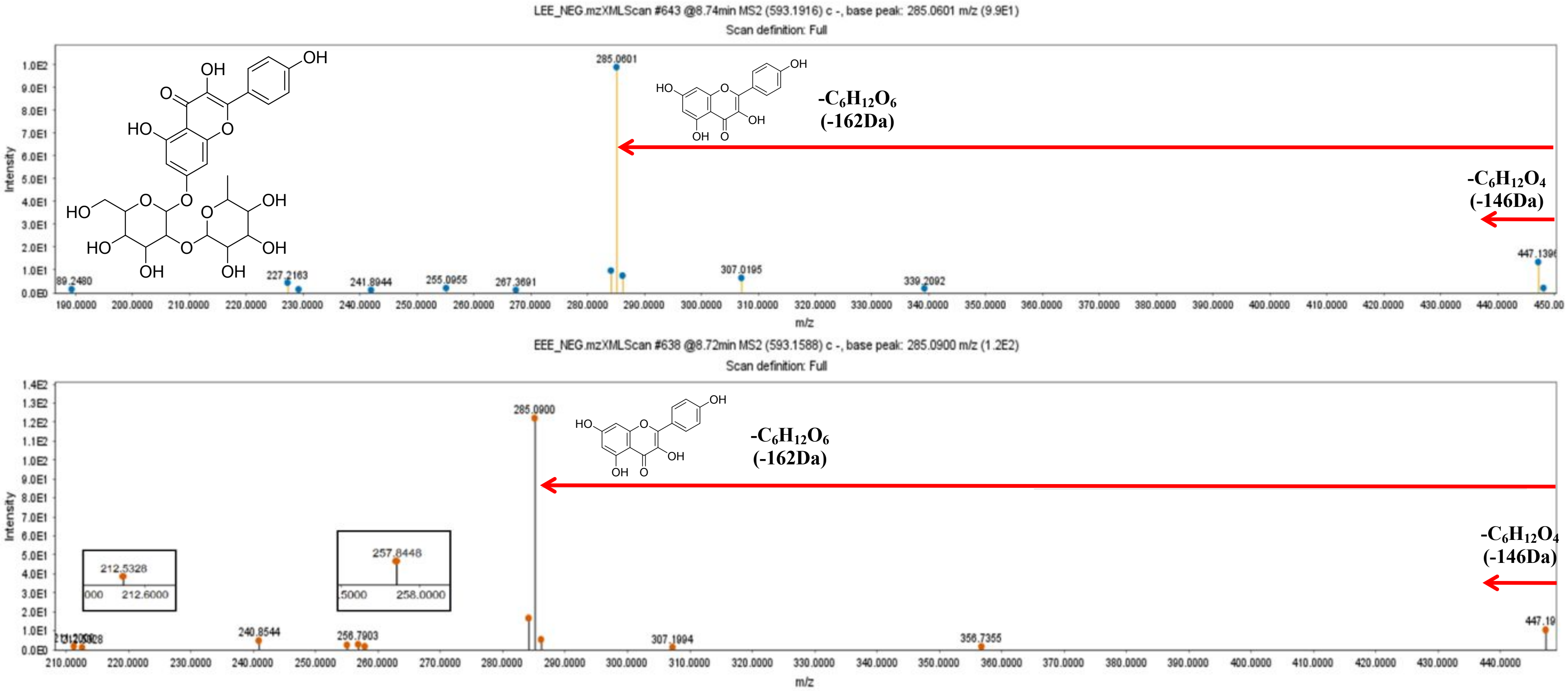

Figure S15. Vicenin-II MS/MS spectrum *m/z* 593 [M-H]$^-$ EEE extract

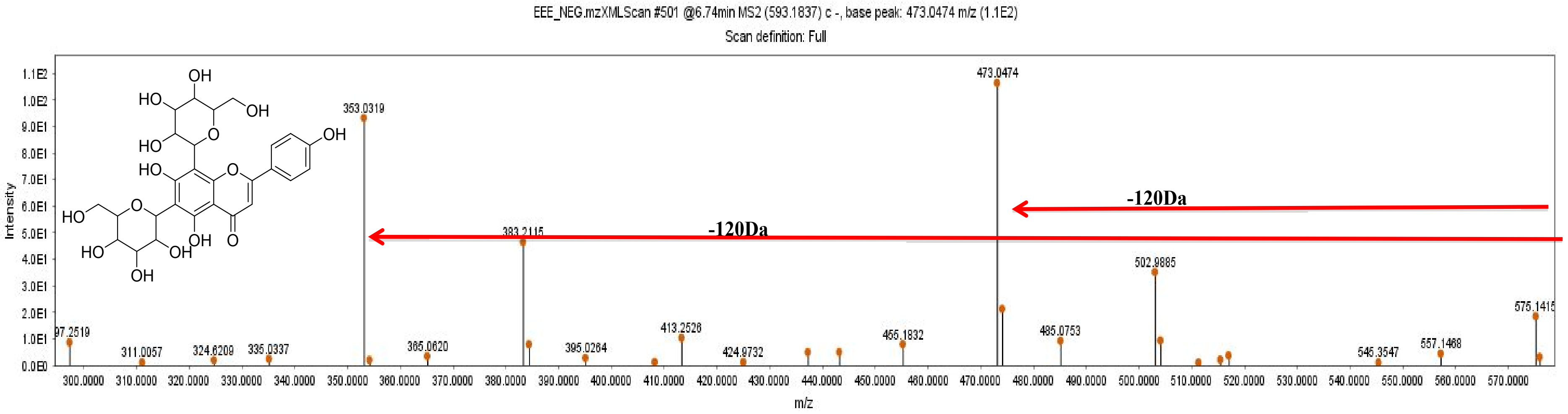

Figure S16. Acacetin MS/MS spectrum *m/z* 283 $[M-H]^-$ LEE and EEE extracts

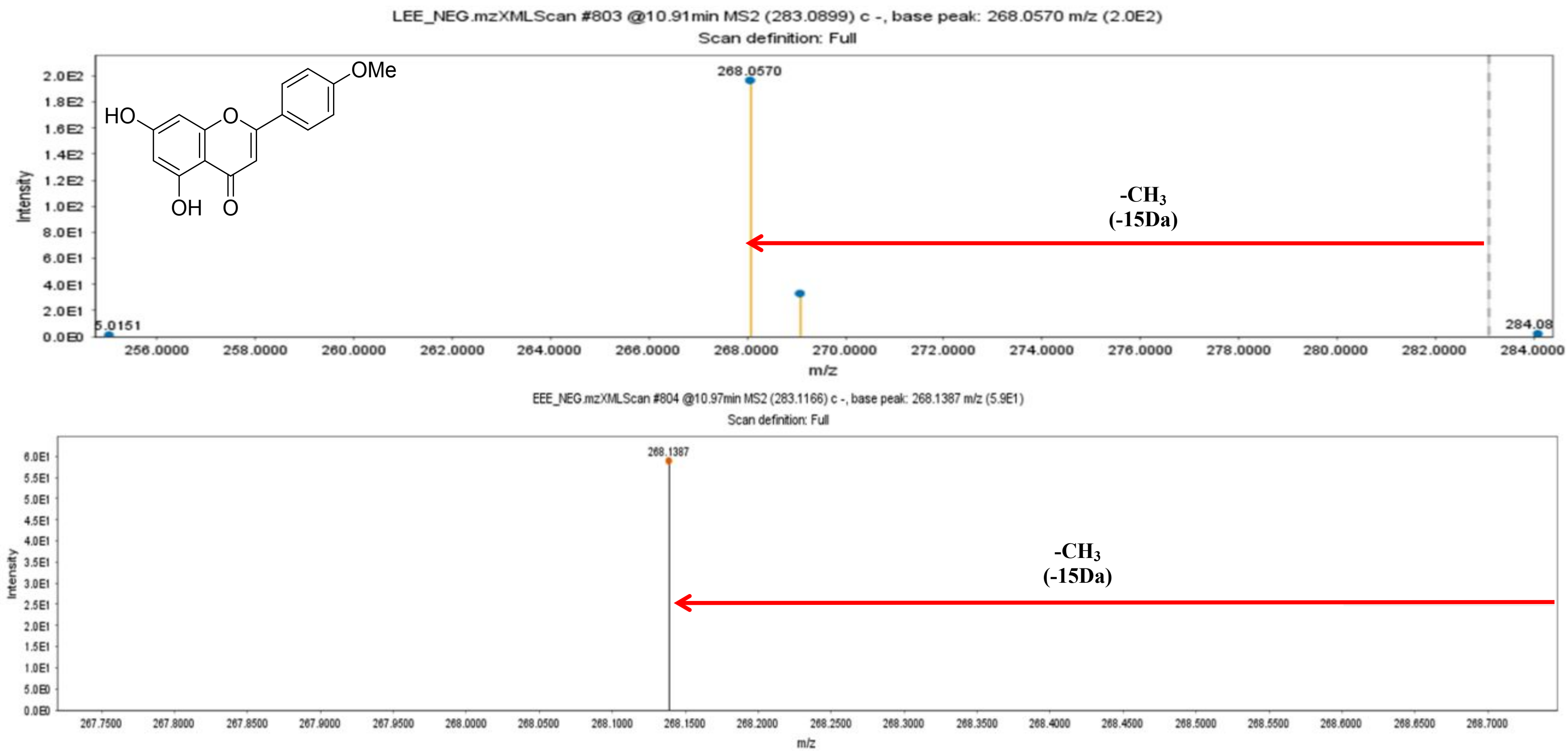

Figure S17. Luteolin MS/MS spectrum *m/z* 285 $[M-H]^-$ LEE and EEE extracts

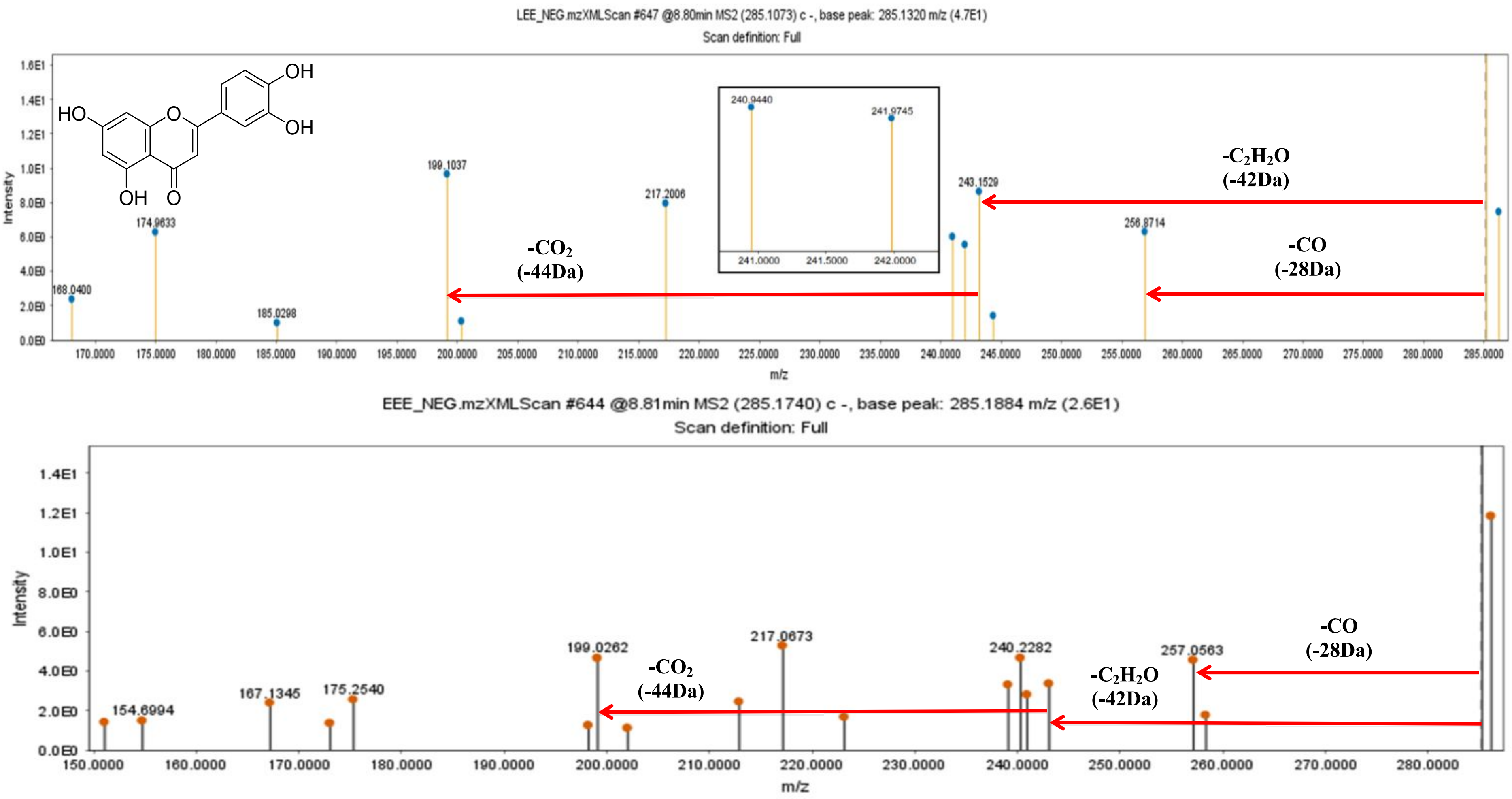

Figure S18. Di-*O*-caffeoylquinic acid MS/MS spectrum *m/z* 515 $[M\text{-}H]^-$ LEE and EEE extracts

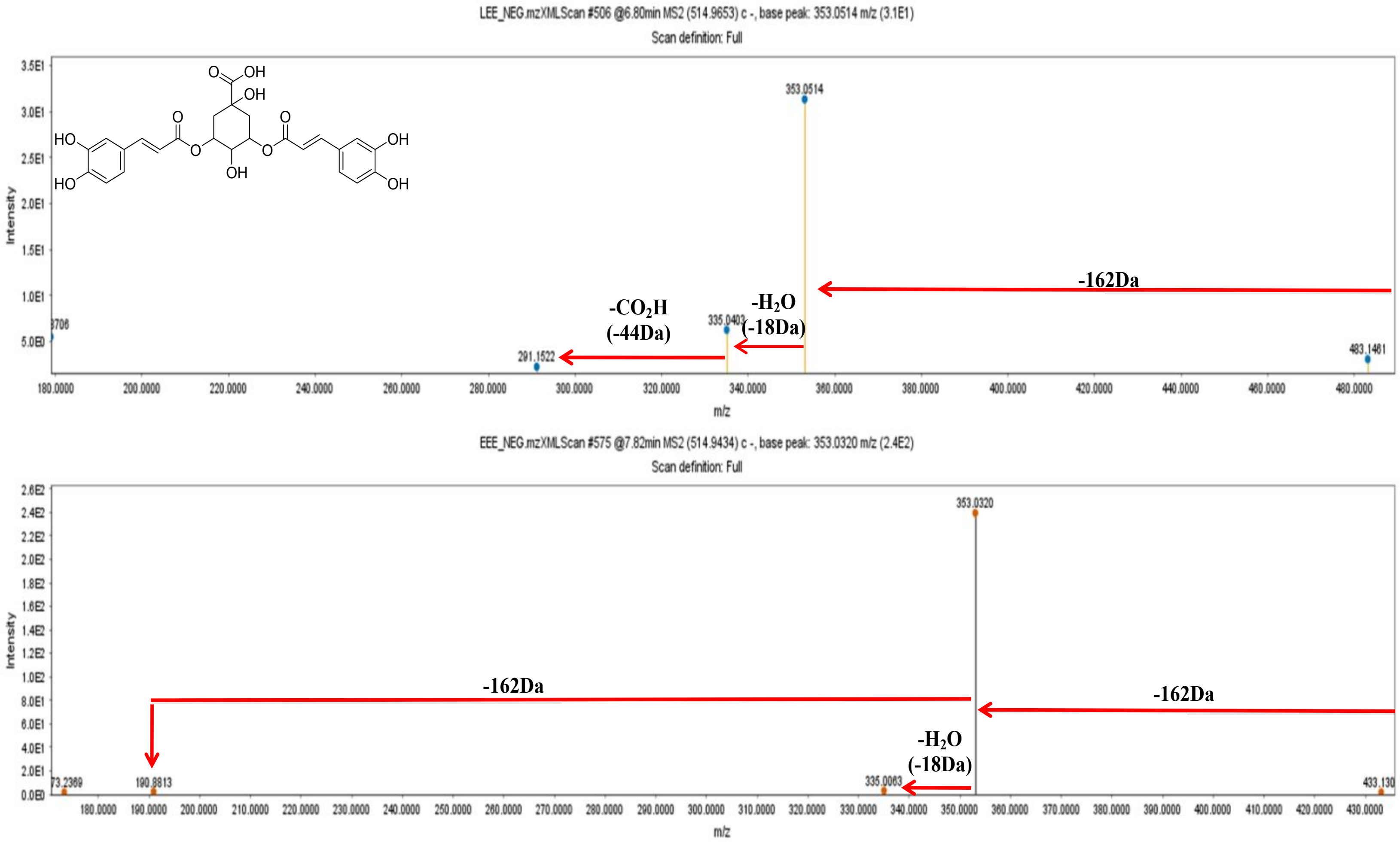

Figure S19. ^{1}H NMR spectrum of friedelin in $CDCl_3$

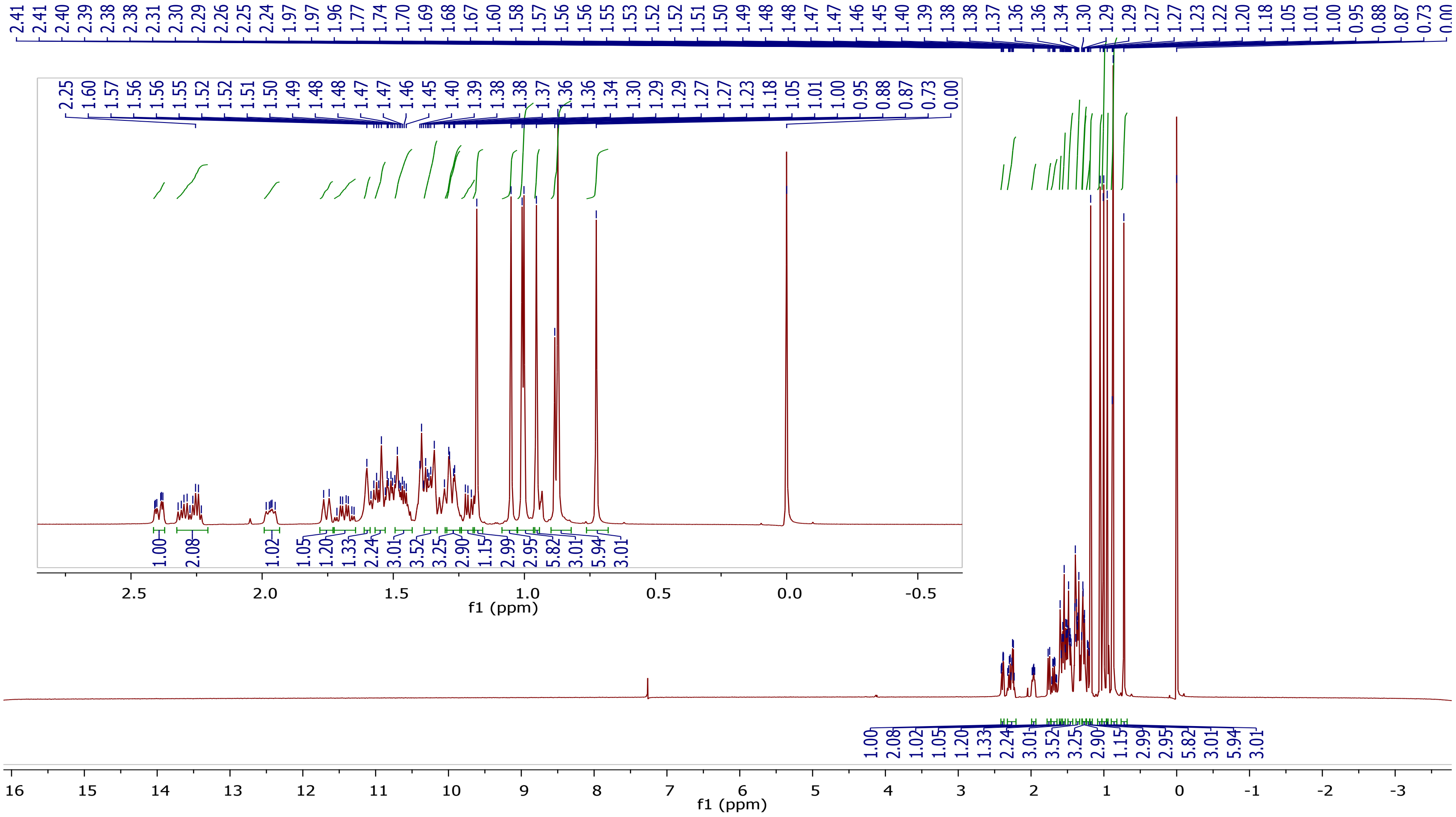

Figure S20. ^{13}C NMR spectrum of friedelin in $CDCl_3$

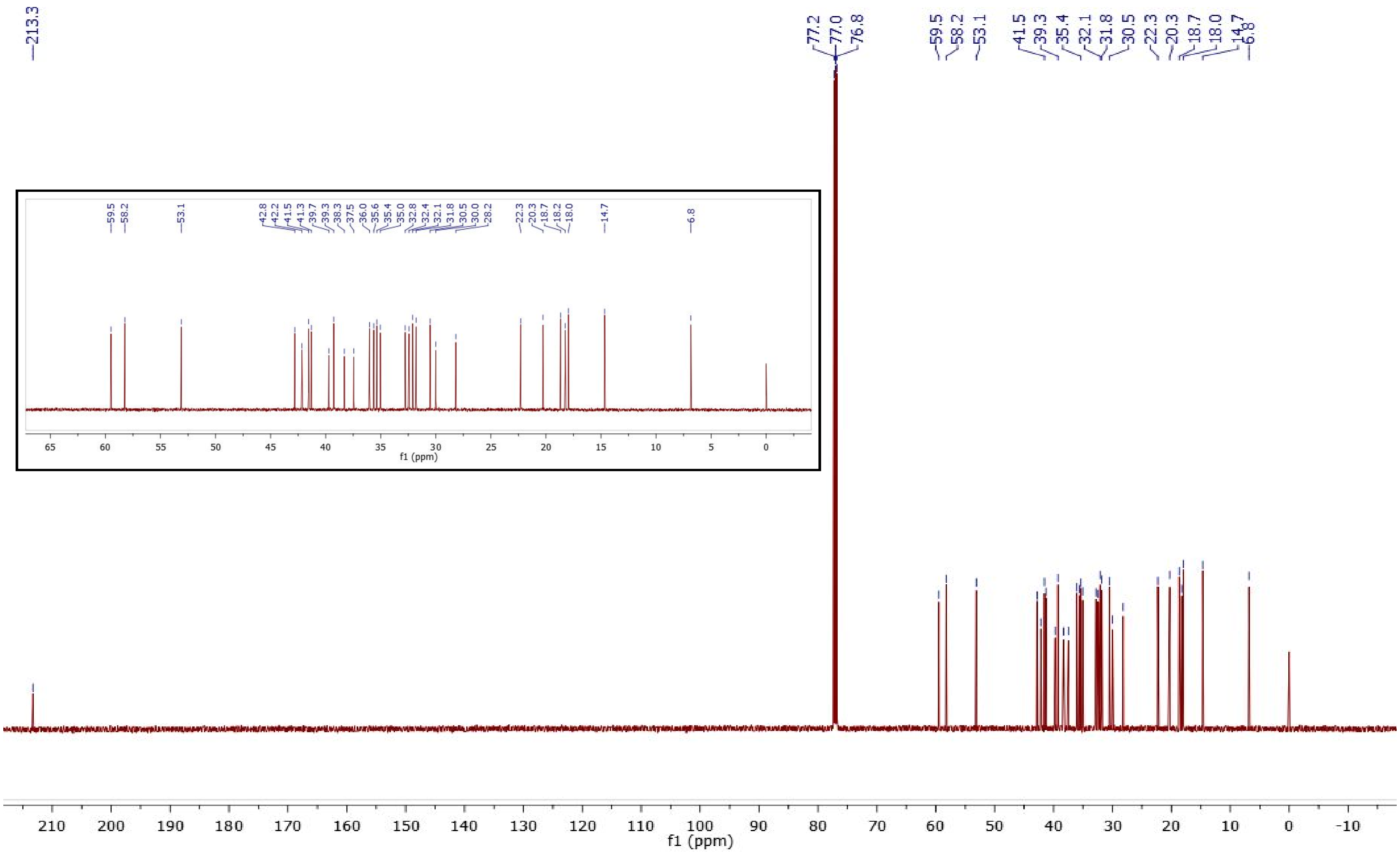

Figure S21. ^{1}H NMR spectrum of betulinic acid in DMSO-d_6

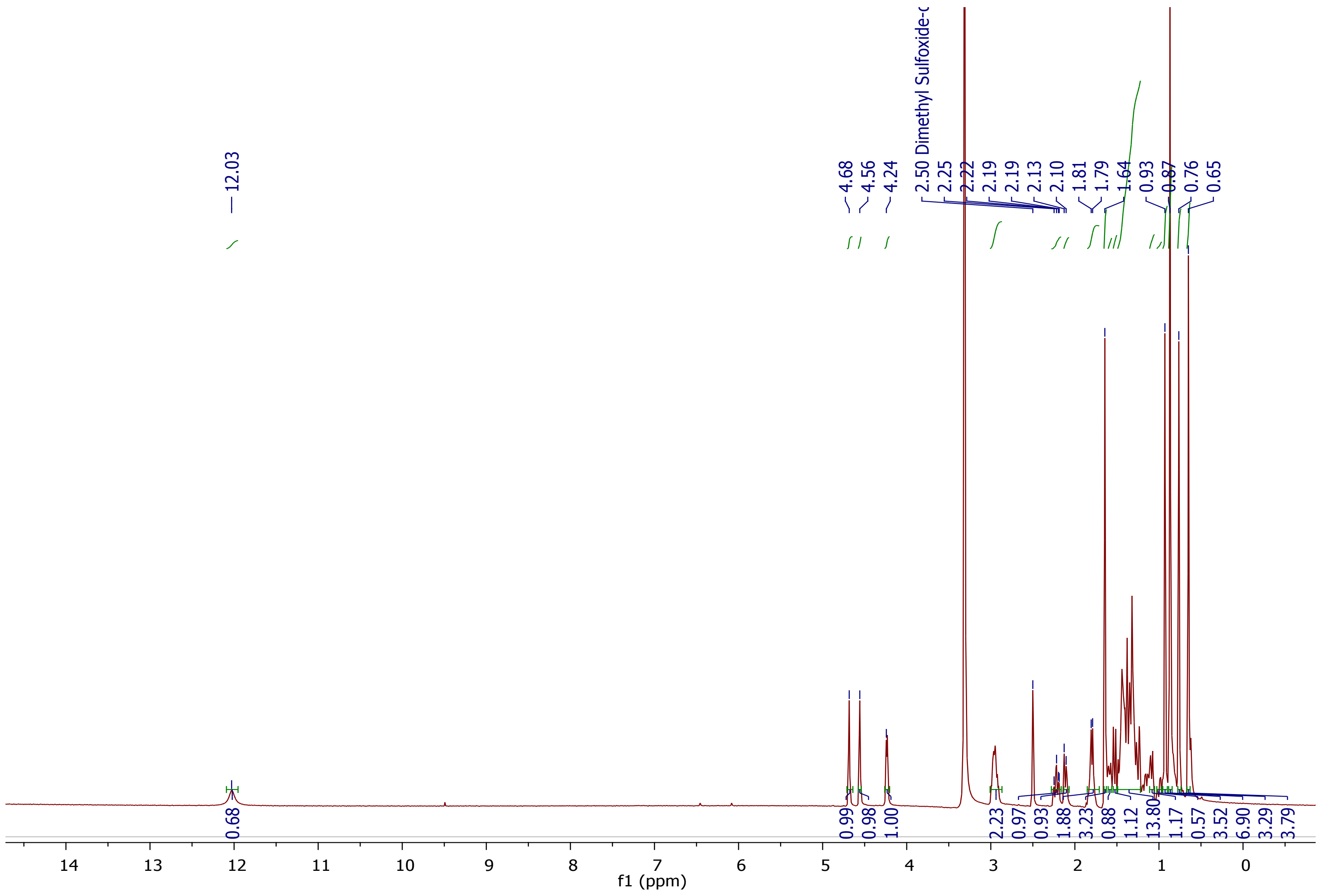

Figure S22. ^{1}H NMR spectrum of betulinic acid with added D_2O

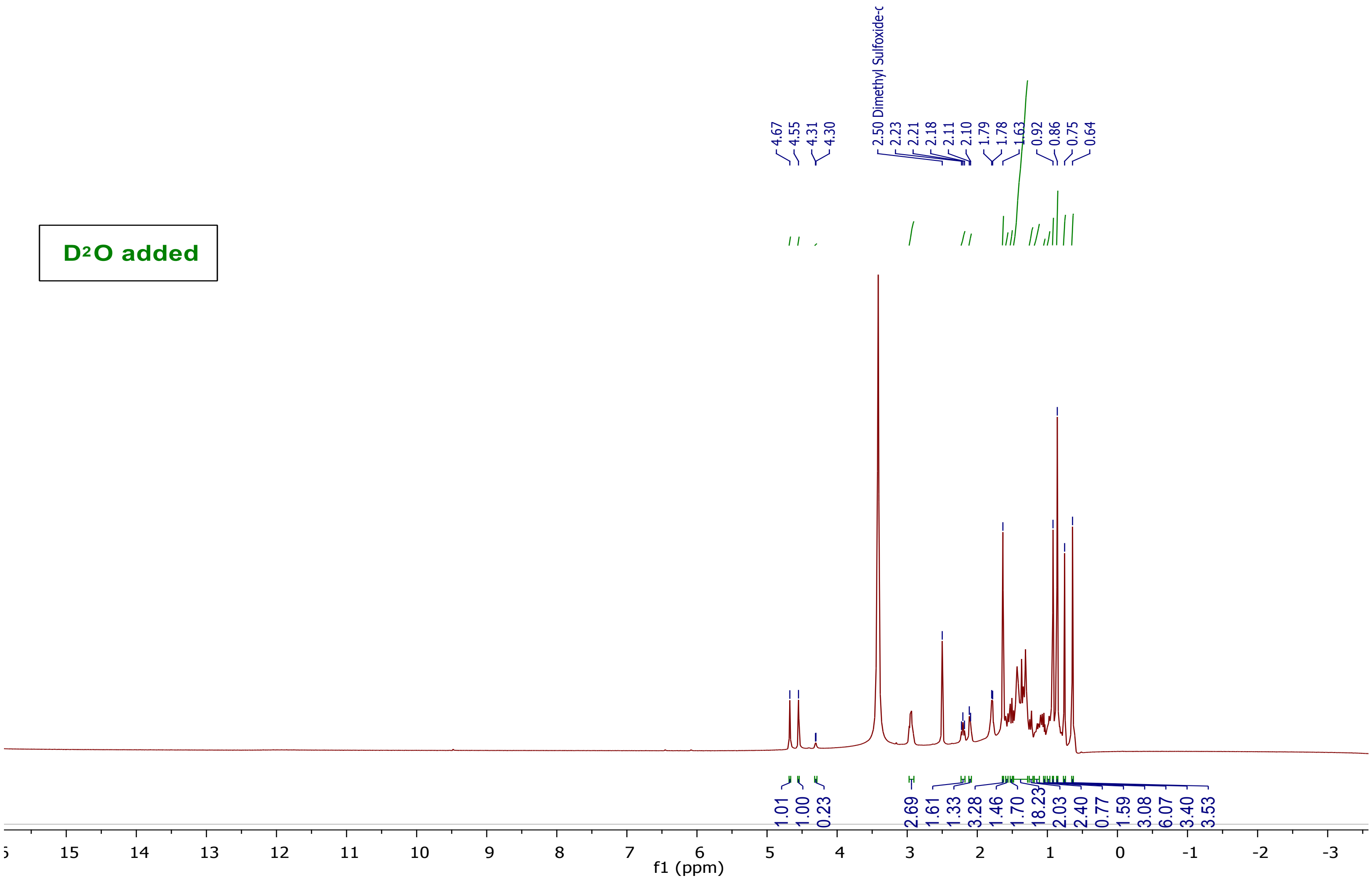

Figure S23. Betulinic acid hydroxyl hydrogen exchange with D_2O solvent

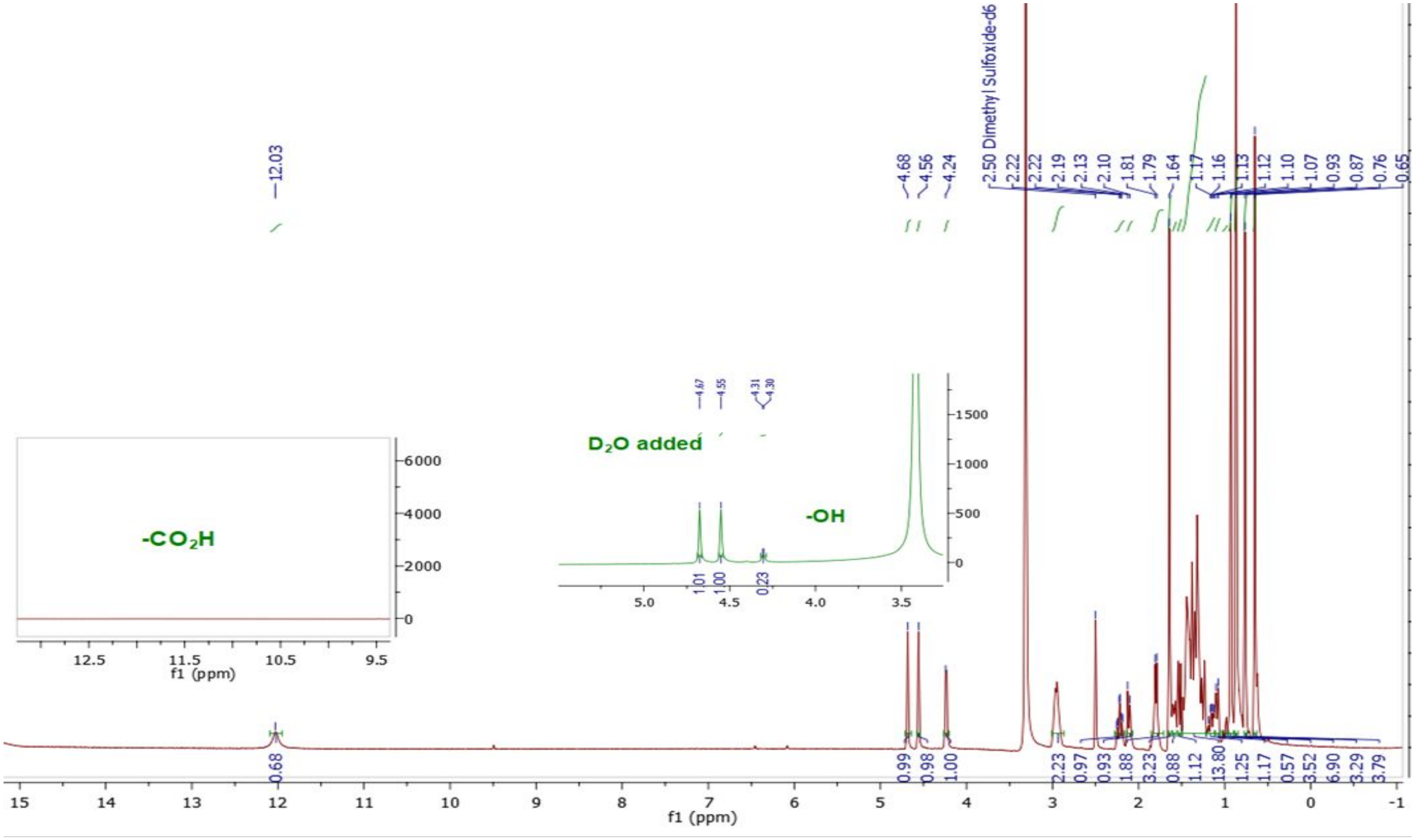

Figure S24. ^{13}C NMR spectrum of betulinic acid in DMSO-d_6

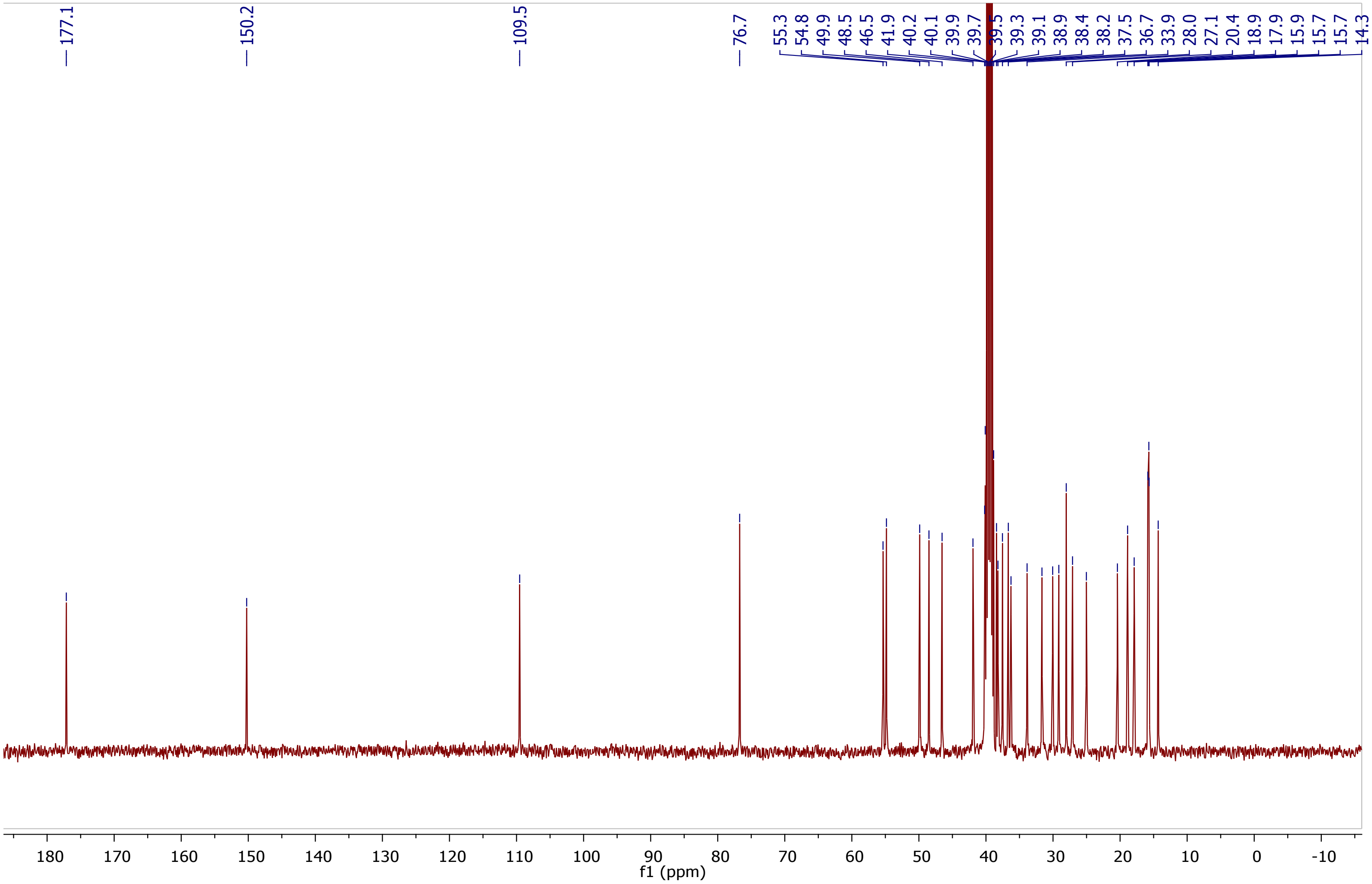

Figure S25. DEPT-135 NMR spectrum of betulinic acid in DMSO-d_6

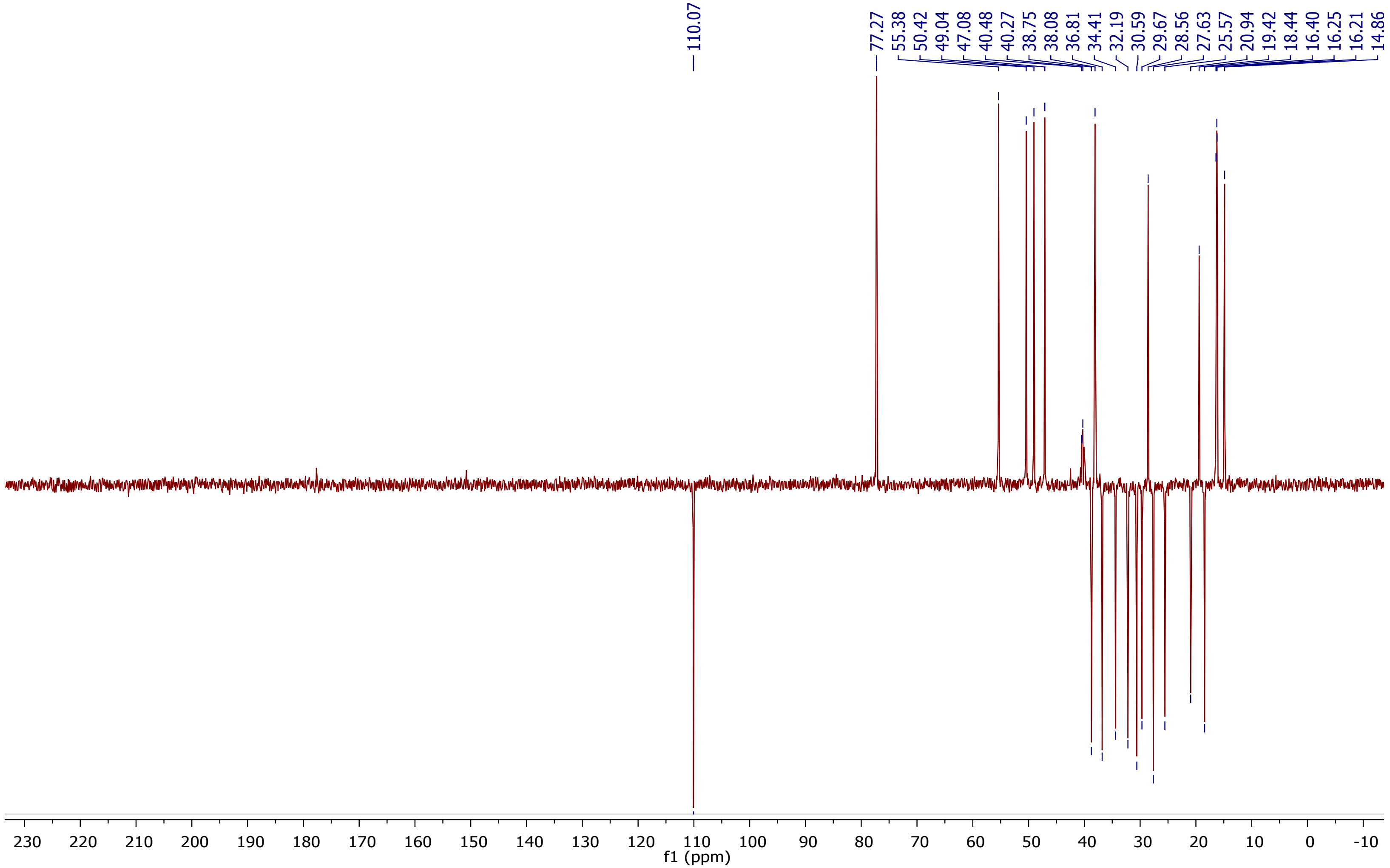

Figure S26. ^{1}H NMR spectrum of acacetin in DMSO-d_6

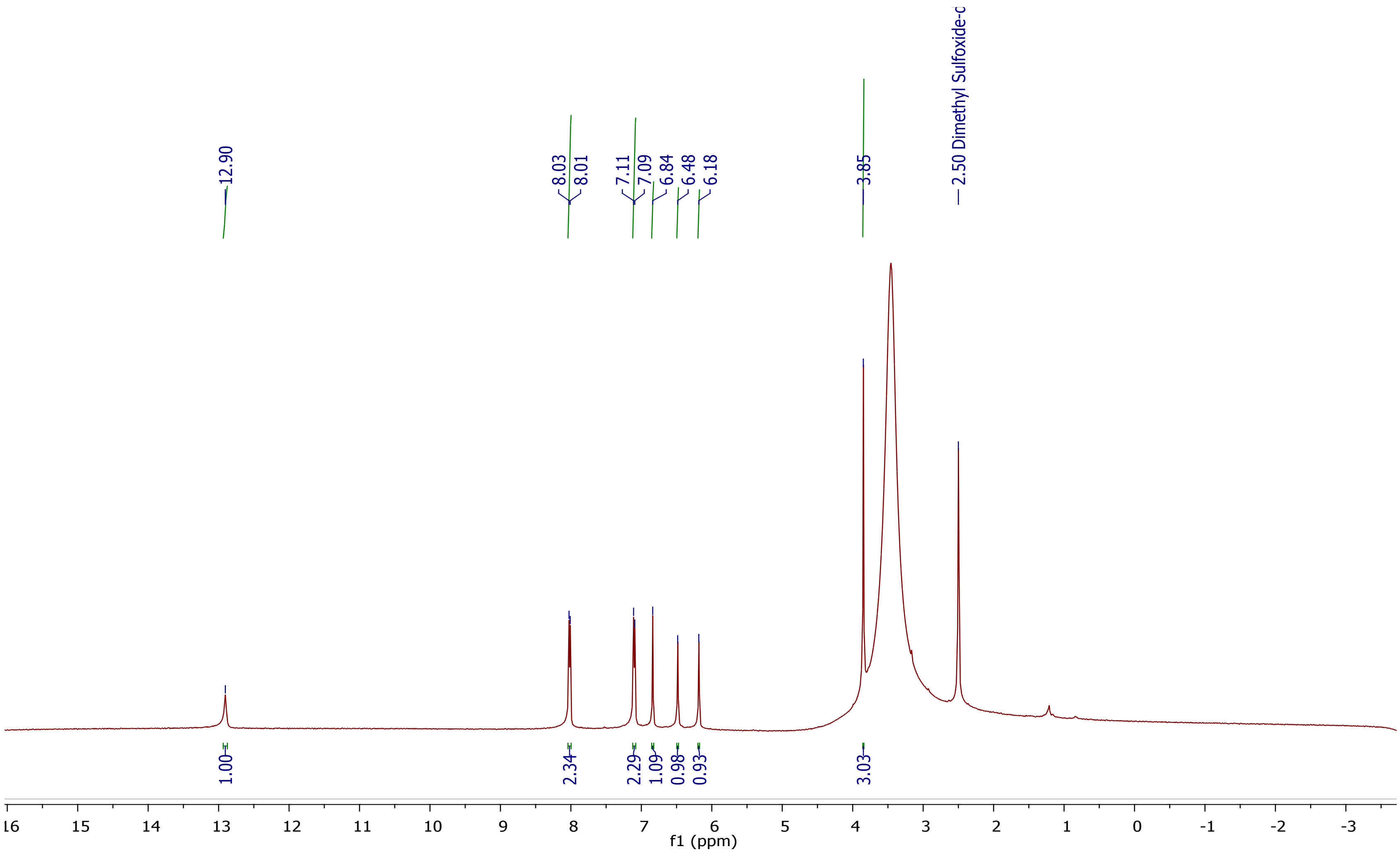

Figure S27. ^{13}C NMR spectrum of acacetin in DMSO-d_6

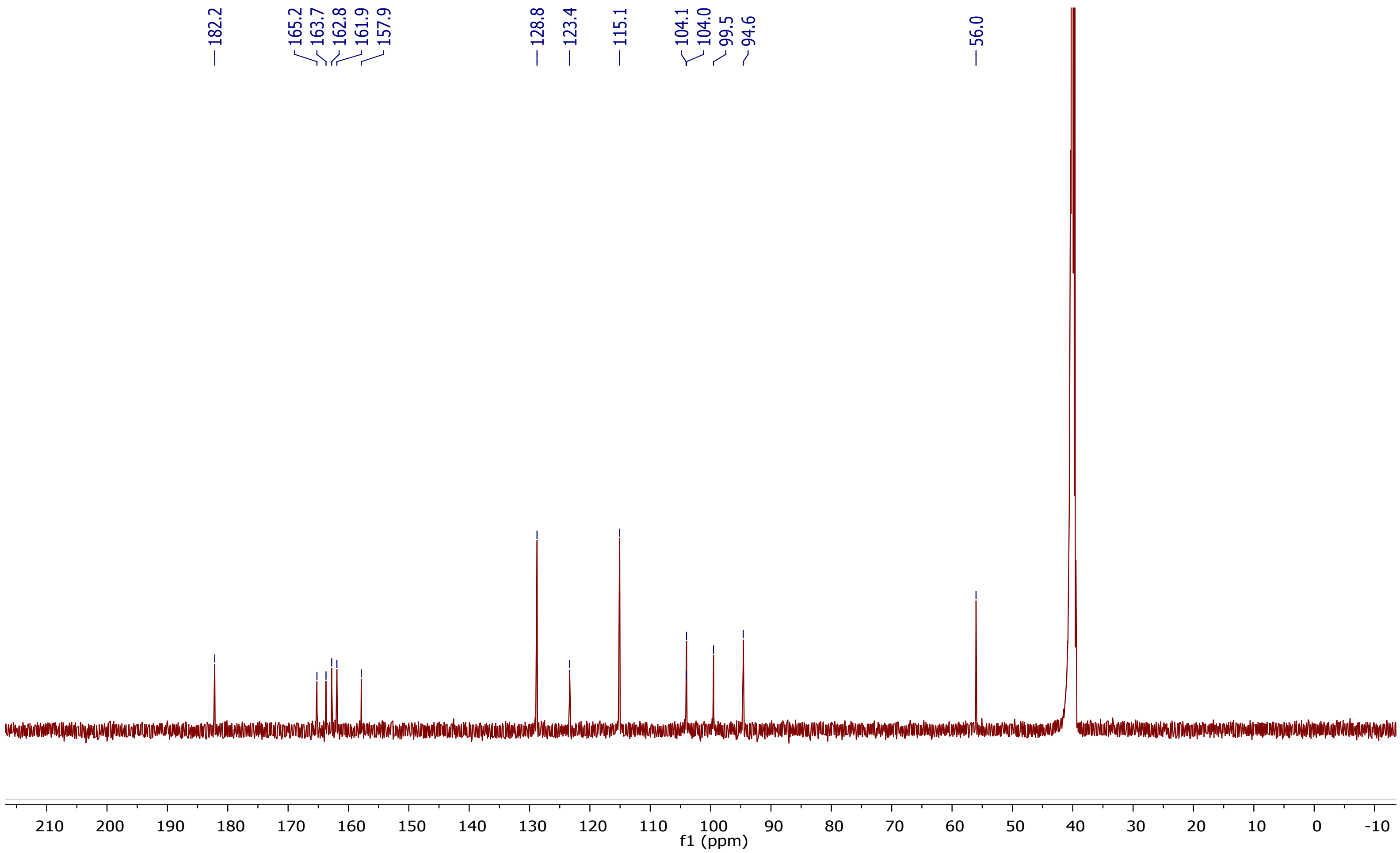

Supplement: Supplementary file 1 [file ao5c03005_si_001.pdf]
